# Phylogenomic analyses and distribution of terpene synthases among *Streptomyces*

**DOI:** 10.3762/bjoc.15.115

**Published:** 2019-05-29

**Authors:** Lara Martín-Sánchez, Kumar Saurabh Singh, Mariana Avalos, Gilles P van Wezel, Jeroen S Dickschat, Paolina Garbeva

**Affiliations:** 1 Department of Microbial Ecology, Netherlands Institute of Ecology (NIOO-KNAW), Droevendaalsesteeg 10, 6708 PB Wageningen, The Netherlands; 2 College of Life and Environmental Sciences, Biosciences, University of Exeter, Penryn Campus, Penryn, Cornwall TR10 9FE, United Kingdom; 3 Institute of Biology, Leiden University, Sylviusweg 72, 2333 BE Leiden,The Netherlands; 4 University of Bonn, Kekulé-Institute of Organic Chemistry and Biochemistry, Gerhard-Domagk-Straße 1, 53121 Bonn, Germany

**Keywords:** biosynthesis, evolution, geosmin, *Streptomyces*, terpenes

## Abstract

Terpene synthases are widely distributed among microorganisms and have been mainly studied in members of the genus *Streptomyces*. However, little is known about the distribution and evolution of the genes for terpene synthases. Here, we performed whole-genome based phylogenetic analysis of *Streptomyces* species, and compared the distribution of terpene synthase genes among them. Overall, our study revealed that ten major types of terpene synthases are present within the genus *Streptomyces*, namely those for geosmin, 2-methylisoborneol, *epi*-isozizaene, 7-*epi*-α-eudesmol, *epi*-cubenol, caryolan-1-ol, cyclooctat-9-en-7-ol, isoafricanol, pentalenene and α-amorphene. The *Streptomyces* species divide in three phylogenetic groups based on their whole genomes for which the distribution of the ten terpene synthases was analysed. Geosmin synthases were the most widely distributed and were found to be evolutionary positively selected. Other terpene synthases were found to be specific for one of the three clades or a subclade within the genus *Streptomyces*. A phylogenetic analysis of the most widely distributed classes of *Streptomyces* terpene synthases in comparison to the phylogenomic analysis of this genus is discussed.

## Introduction

*Streptomyces* are soil bacteria that belong to the order of actinomycetales and are a rich source of natural products with broad biotechnological interest. Species of this genus have a remarkable genetic potential to produce a large variety of secondary metabolites with different functions including antibiotics, antifungals, pigments or immunosuppressants [1–3]. These are compounds of diverse chemical nature such as polyketides, peptides, aminoglycosides or terpenoids [4–5].

Terpenoids are the largest and the most diverse class of natural compounds known to date and include the initial products of terpene synthases and all derivatives made from them in tailoring steps. This very diverse class of organic compounds is best known as plant metabolites. However, recent studies revealed that terpenoids can be produced by all kingdoms of life including bacteria, fungi and protists [6–10]. The ability of an organism to produce terpenoids relies on whether the organism contains terpene synthase genes. Biosynthetically, the production of the different types of terpenes depends on the precursors that these synthases can accommodate: geranyl diphosphate (monoterpenes, C10), farnesyl diphosphate (sesquiterpenes, C15) and geranylgeranyl diphosphate (diterpenes, C20). The biological function of terpenes is best studied for plants where they play important roles in aboveground plant–insect, plant–pathogen and plant–plant interactions [11]. However, terpenes might also play important roles in belowground interspecific interactions [12]. Terpene synthases are in fact widely distributed among soil microorganisms, and they have been mainly studied in *Streptomyces* species [9,13]. Some volatile terpenes, such as geosmin and 2-methylisoborneol (2-MIB), responsible for the smell of wet soil after rain, have been known for a long time to be produced by *Streptomyces* species [14–15]. Many terpene synthases from *Streptomyces* have been studied and characterised [13]. However, little is known about the distribution of terpene synthase encoding genes among *Streptomyces*. Are terpene synthase genes specific for certain species or randomly distributed among *Streptomyces*?

To address this question, phylogenomic analyses of *Streptomyces* species were performed, using complete genomes available in the NCBI database and compared the distribution of terpene synthase genes among them. Furthermore, we studied whether phylogenetic trees calculated based on the three most abundant terpene synthases in *Streptomyces* represent the evolution of the *Streptomyces* species based on the whole genome-based phylogenetic analyses.

## Results and Discussion

### Whole genome-based phylogenetic analyses of *Streptomyces* species

**Figure 1:**
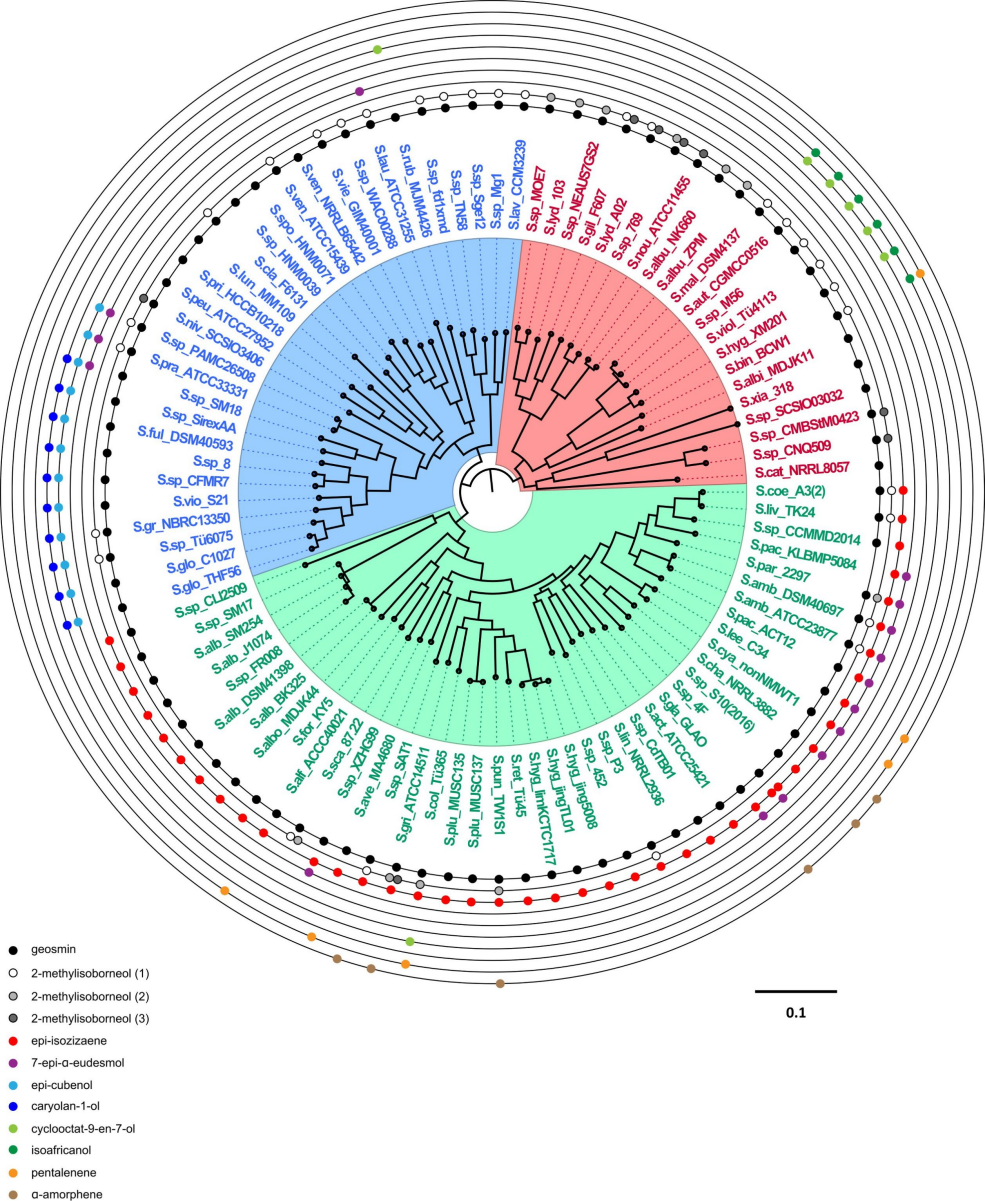
Whole-genome phylogenetic analyses of *Streptomyces* species. Rooted maximum likelihood phylogeny of 93 *Streptomyces* species with fully sequenced genomes based on 575 conserved single copy orthologues. The species separated in three main groups are indicated by different colour-shaded areas. The outer rings show the distribution of different types of terpene synthases in the *Streptomyces* species. Another version of this tree using 5 non-*Streptomyces* species as outgroups can be found in the Figure S1 in Supporting Information File 1. The GenBank accession numbers of the sequences are provided in Table S2 (Supporting Information File 1).

Genome sequences from 93 *Streptomyces* species for which a complete genome was available (represented by a single scaffold and a complete list of annotated protein sequences), were selected to construct a whole genome-based phylogenetic tree (Figure 1). The NCBI database was accessed on September 30th 2018. An orthologues-based approach was adopted to generate a species tree using OrthoFinder. OrthoFinder resulted in a total of 19980 orthologue groups (Table S1, Supporting Information File 1). A total number of 575 single copy orthologues were further selected for the generation of the species tree. Based on these phylogenetic analyses, the *Streptomyces* species clustered in three different clades (indicated in blue, green and red in Figure 1). This separation into three different clades agrees with the study previously reported by McDonald and Currie, 2017 [16]. Based on phylogenetic analyses of 94 housekeeping genes, they showed a separation of *Streptomyces* species in two major clades and a third group of other lineages.

### Distribution of terpene synthases in *Streptomyces*

**Figure 2:**
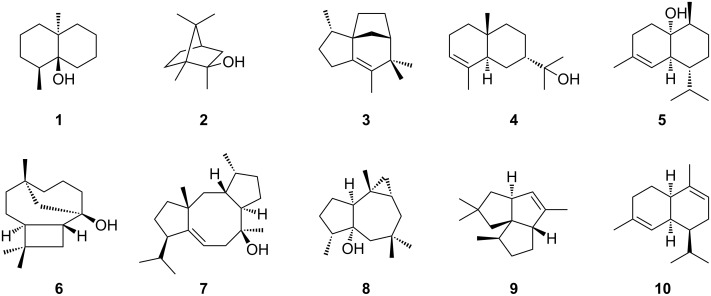
Structures of the products of the ten most abundant terpene synthases in *Streptomyces*.

We analysed the distribution of the different types of terpene synthases among *Streptomyces* species with complete genomes. Besides a few rare terpene synthases only occuring in a few or a single species, ten major types of terpene synthases were present among these *Streptomyces* species, including the terpene synthases for geosmin (**1**), 2-methylisoborneol (2-MIB) (**2**), *epi*-isozizaene (**3**), 7-*epi*-α-eudesmol (**4**), *epi*-cubenol (**5**), caryolan-1-ol (**6**), cyclooctat-9-en-7-ol (**7**), isoafricanol (**8**), pentalenene (**9**) and α-amorphene (**10**) (Figure 1 and Figure 2).

The geosmin synthases were the most widely distributed, as they were present in all except one of the *Streptomyces* species (*S. pactum* KLBMP 5084) (Figure 1). This finding suggests that geosmin may have an important ecological function as a chemical signal or as protective specialised metabolite against biotic and abiotic stresses, similarly as the roles played by terpenoids in plants [11]. However, although geosmin was discovered more than 50 years ago [14], its biological or ecological function still remains unclear. *Streptomyces pactum* KLBMP 5084 (the only species included in this study that does not carry geosmin synthases) is an endophytic plant growth-promoting bacterium that provides salt tolerance to the halophytic plant *Limonium sinense* (Plumbaginaceae) [17]. The absence of a geosmin synthase in this bacterium leads us to hypothesise that the role of geosmin may be complemented by the plant host. The only other plant endophyte among the 93 species is *Streptomyces* sp. SAT1 (see Table S9 (Supporting Information File 1) for a list of the isolation sources and habitats of the 93 strains). This strain is an endophyte of the flowering plant *Adenophora trachelioides* from the Campanulaceae family and it does contain a copy of *geoA*, the gene encoding for geosmin synthase. Some species such as *Streptomyces* sp. SirexAA-E harbour a silent geosmin synthase encoding gene in their genomes and do not produce this degraded sesquiterpene under laboratory culture conditions [9]. It will therefore be interesting to investigate whether the geosmin synthase in *Streptomyces* sp. SAT1 is expressed, and to further determine the role of terpenoids in the endophytic life style.

**Scheme 1:**
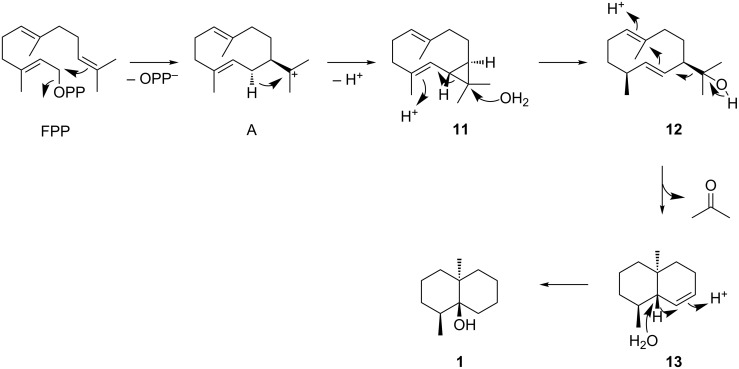
Mechanism for the cyclisation of FPP to geosmin.

The first geosmin synthase was characterised from *Streptomyces coelicolor* [18]. Geosmin synthases are composed of two domains that both exhibit the typical highly conserved motifs of type I terpene synthases, including the aspartate-rich motif, the NSE triad, the pyrophosphate sensor and the RY pair [19–21]. Both domains have a catalytic activity, the N-terminal domain for the conversion of FPP into the intermediate sesquiterpene alcohol (1(10)*E*,5*E*)-germacradien-11-ol (**12**), and the C-terminal domain for its further conversion into geosmin with cleavage of **12** into acetone and the octalin **13** through a retro-Prins fragmentation (Scheme 1) [22–24]. The proposed neutral intermediate isolepidozene (**11**) has so far only been reported from the S233A enzyme variant of geosmin synthase from *S. coelicolor* [18].

**Scheme 2:**
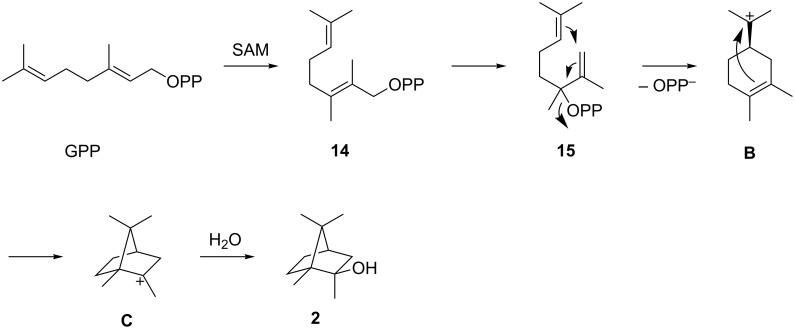
Biosynthesis of 2-MIB (**2**). First, GPP is methylated to **14** by a SAM-dependent methyltransferase, followed by a terpene synthase catalysed cyclisation through a cationic cascade to **2**.

The second most widely distributed terpene synthases are the 2-MIB synthases (Figure 1). As discussed below, the phylogenetic analysis of 2-MIB synthases classifies these enzymes into three different groups. This distribution is also indicated in Figure 1 (white, light gray and dark gray circles). The 2-MIB synthases are present in members of all the three clades from the whole genome phylogenetic tree (Figure 1), but are most abundant in members of the clade depicted in red. These terpene synthases catalyse a unique cyclisation reaction utilizing the modified substrate 2-methyl-GPP to form 2-MIB (**2**) [25–26]. An *S*-adenosylmethionine (SAM) dependent methyl transferase is responsible for the methylation of GPP into 2-methyl-GPP (**14**, Scheme 2). Its isomerisation to **15** allows for a cyclisation via the cationic intermediates **B** and **C** to **2**. Genes encoding for SAM-dependent methyl transferases were found forming a cluster together with the 2-MIB synthase in several *Streptomyces* species [26–27]. Besides the C-terminal domain typical of class I terpene synthases, these enzymes contain an additional proline-rich N-terminal domain that appears to be disordered in the crystal structure of 2-MIB synthase. The function of this domain is unknown, but it is conserved in most 2-MIB synthases and not present in any other terpene synthase [20,28].

**Scheme 3:**
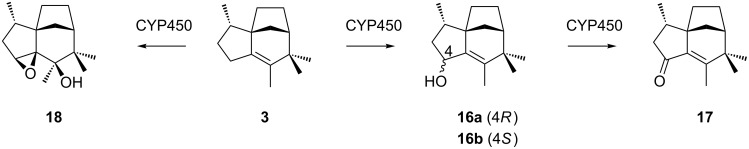
Oxidation products derived from **3** by the cytochrome P450 monooxygenase that is genetically clustered with the *epi*-isozizaene synthase in streptomycetes.

*epi*-Isozizaene (**3**) is a tricyclic sesquiterpene precursor of the antibiotic albaflavenone (**17**) (Scheme 3) [29]. Furthermore, both enantiomers of the corresponding alcohols (*R*)- and (*S*)-albaflavenol (**16ab**) and the epoxide 4β,5β-epoxy-2-*epi*-zizaan-6β-ol (**18**) are known oxidation products that are all made by a cytochrome P450 monooxygenase [10,29] that is genetically clustered with the *epi*-isozizaene synthase for the cyclisation of FPP to **3** [30]. These enzymes are the most widespread sesquiterpene synthases in bacteria, and their coding genes are present in the genomes of more than 100 of the sequenced *Streptomyces* species [13]. Interestingly, *epi*-isozizaene synthases are only present in members of one clade (indicated as the green clade) in the phylogenetic analyses shown in Figure 1 and occur in almost all species of this clade with one exception (*S. scabiei* 87.22), suggesting an (unknown) ecological function of **3** or one of its oxidation products for streptomycetes of this clade for their adaption to a specific ecological niche.

7-*epi*-α-Eudesmol (**4**) synthases are mostly present in a small group of species within the phylogenomic clade depicted in green in Figure 1, with some exceptions (*S. laurentii* ATCC 31255, *Streptomyces* sp PAMC 26508, *S. pratensis* ATCC 33331, *Streptomyces* sp_SM18 and *Streptomyces* sp. XZHG99, Figure 1). These exceptions may indicate horizontal gene transfer of the genes encoding for these enzymes. The sesquiterpene 7-*epi*-α-eudesmol synthase from *S. viridochromogenes* DSM 40736 has been chemically characterised in vivo by heterologous expression in *E. coli* BL21 and identification of the product in culture headspace extracts by GC–MS [31]. Compound **4** was also isolated from in vitro incubations of FPP with the recombinant enzyme and its optical rotation was shown to be opposite to the material from *Eucalyptus* [32], but the absolute configuration remains unknown. Production of this sesquiterpene by *S. viridochromogenes* DSM 40736 has not been observed [31], but **4** was occassionally reported from other streptomycetes encoding a 7-*epi*-α-eudesmol synthase [33–34].

*epi*-Cubenol (**5**) and caryolan-1-ol (**6**) synthases almost always occur together in one strain. We found only two examples of a strain that has a gene for caryolan-1-ol synthase but not for *epi*-cubenol synthase. These enzymes were found only in a sub-branch of closely related *Streptomyces* species from the blue clade and not present in members of any other phylogenomic group (Figure 1). Both enzymes have been identified and characterised in *S. griseus* NBRC 13350 [35–36] and their enzymatic mechanisms for the cyclisation of FPP have been investigated [35,37–39].

**Scheme 4:**
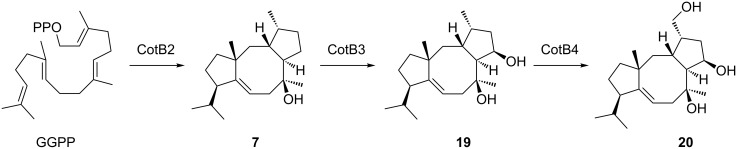
Biosynthesis of cyclooctatin (**20**) from **7**.

Cyclooctat-9-en-7-ol (**7**) and isoafricanol (**8**) synthases are mainly characteristic for a group of very closely related species in the phylogenomic clade depicted in red in Figure 1, with two exceptions, *S. rubrolavendulae* MJM4426 and *S. collinus* Tü 365, members of the other two phylogenomic clades that also present a cyclooctat-9-en-7-ol synthase. Cyclooctat-9-en-7-ol synthase (CotB2) from *S. melanosporofaciens* was the first bacterial type I diterpene cyclase characterised [40] and its crystal structure was the first of a diterpene cyclase of bacterial origin reported [41]. Isoafricanol synthases were first noticed in *S. violaceusniger* and *S. rapamycinicus* based on the presence of **8** in culture headspace extracts as a major sesquiterpene [34,42], followed by the biochemical characterisation of the recombinant enzyme from *Streptomyces malaysiensis* [43]. The diterpene **7** is a precursor to the lysophospholipase inhibitor cyclooctatin (**20**) formed by the action of two genetically clustered cytochrome P450 monooxygenases CotB3 and CotB4 (Scheme 4) [40,44], while no derivatives from **8** are currently known.

Pentalenene (**9**) and α-amorphene (**10**) synthases are the least abundant terpene synthases in *Streptomyces* species, each present in only 6 species (Figure 1). They are mostly present in members of the phylogenomic clade depicted in green in Figure 1, except for one case, *S. bingchenggensis* BCW1, but within the green clade their distribution is scattered and the number of identified genes for these enzymes is too low to draw conclusions on their occurrence in *Streptomyces*. The pentalenene synthase from *S. exfoliatus* was the first characterised bacterial terpene synthase [45–46]. Its crystal structure was also the first reported for a bacterial terpene synthase [47]. Pentalenene synthase catalyses the cyclisation of FPP into pentalenene, which is the first step in the biosynthesis of the antibiotic pentalenolactone. This mechanism has been extensively studied and involves the initial ionisation of the substrate FPP and the formation of a humulyl cation as an intermediate in the biosynthesis of pentalenene [45–46,48–49], while the later steps of the cyclisation cascade were subject to revision based on the findings of quantum chemical calculations [50–51]. The α-amorphene synthase from *S. viridochromogenes* DSM 40736 was characterised by heterologous expression in *E. coli* BL21 [31] and by in vitro experiments with the purified enzyme [32].

### Phylogenetic analysis of geosmin synthases

**Figure 3:**
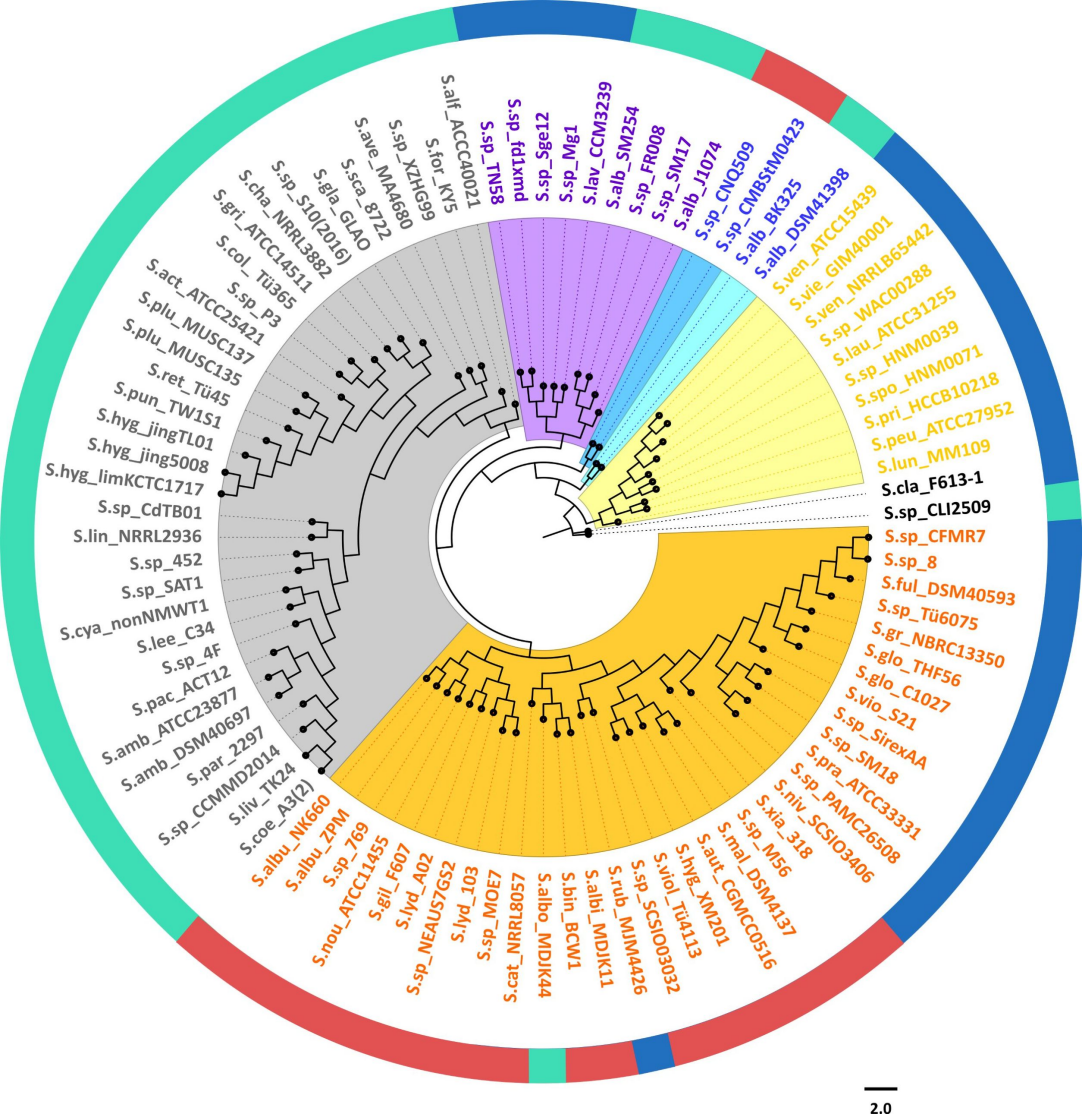
Phylogenetic tree of geosmin synthases. Unrooted maximum likelihood phylogenetic tree of 92 geosmin synthases from the *Streptomyces* species present in the phylogenetic tree in Figure 1. The colours in the outer ring correspond to the colours of the three main phylogenomic groups in the whole-genome species tree and indicate to which phylogenomic group each species belongs. The GenBank accession numbers of the geosmin synthases are listed in Table S3 (Supporting Information File 1).

In order to determine if the geosmin synthases co-evolved with the *Streptomyces* species a phylogenetic tree was constructed with the geosmin synthases of all the species present in the full genome tree. As seen in Figure 3, the geosmin synthases separated into different clades. These clades do not fully correspond with specific phylogenomic groups from the genome-based analyses. Most of the geosmin synthases of the green and red phylogenetic clade in the whole genome-based tree of Figure 1 grouped together into one clade. The enzymes from the blue phylogenetic clade in the genome-based tree were the most scattered. All these results may point to the occurrence of horizontal gene transfer within the genus *Streptomyces*. However, if bacteria from other taxonomic groups such as myxobacteria and cyanobacteria and their geosmin synthases are included in a phylogenetic analysis, it can be seen that the geosmin synthase amino acid sequences from distantly related organisms clearly fall into distant clades [33]. Therefore, these results could also be interpreted as evidence for a rapid evolution of secondary metabolite genes to create new natural products with beneficial ecological functions for the producing organism. While many streptomycetes produce geosmin as a major metabolite of their bouquets of volatiles, the number and amounts of geosmin synthase side products associated with it can vary [33–34], possibly as a result of an evolution of enzyme function.

### Phylogenetic analysis of 2-MIB synthases

**Figure 4:**
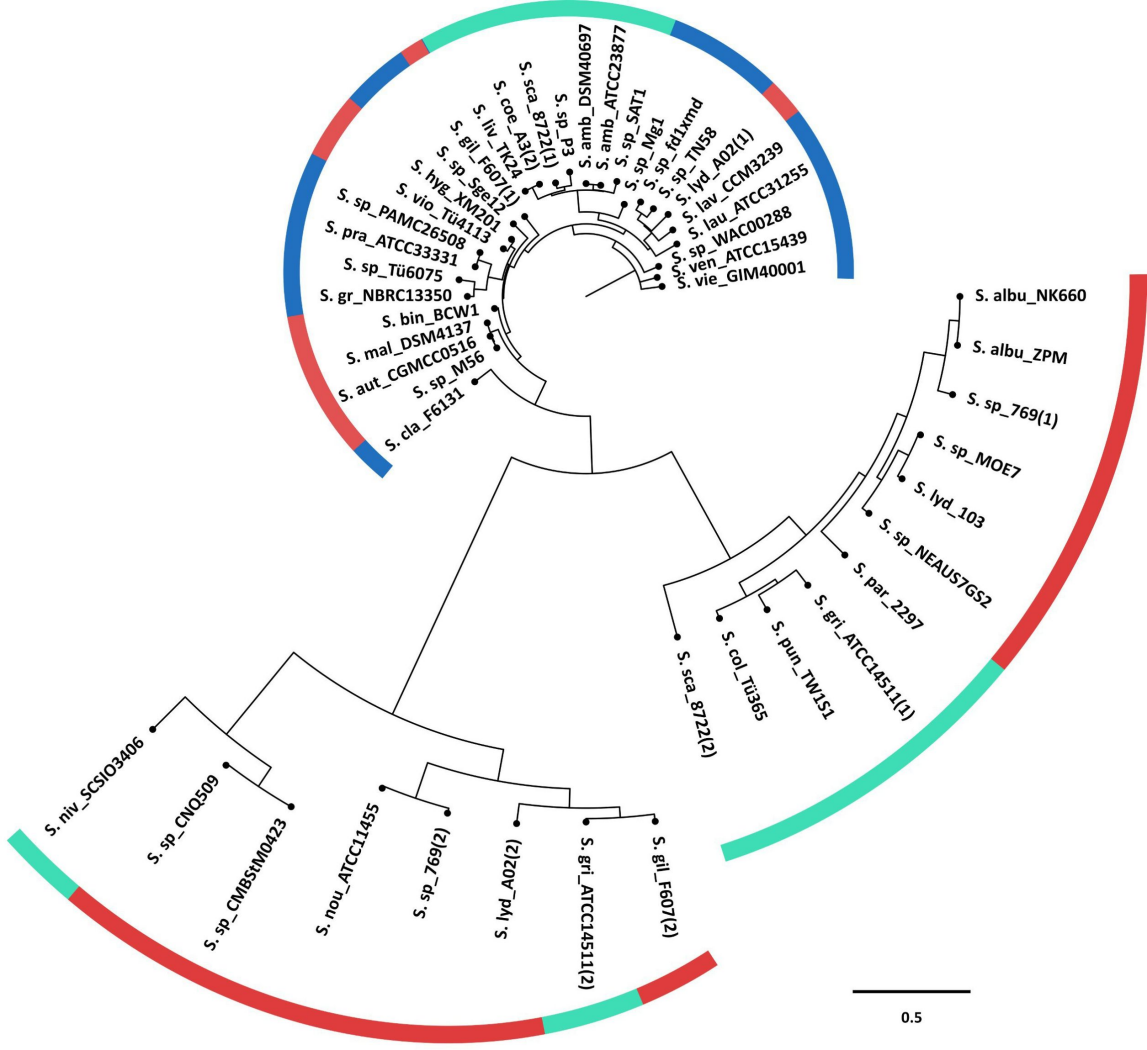
Phylogenetic tree of 2-MIB synthases. Unrooted maximum likelihood phylogenetic tree of 48 2-MIB synthases from the *Streptomyces* species in the phylogenetic tree in Figure 1. The colours of the outer curved lines correspond to the colours of the three main phylogenomic groups in the whole-genome species tree and indicate to which phylogenomic group each species belongs. The GenBank accession numbers of the 2-MIB synthases are listed in Table S4 (Supporting Information File 1).

To gain insights into the evolution of the 2-MIB synthases a phylogenetic analysis of all the enzymes present in the *Streptomyces* species analysed in our study were performed (Figure 4). The phylogenetic tree of the 2-MIB synthases shows a clear separation into three clades (also indicated in Figure 1: group 1, white circles, representing the major clade on the top of Figure 4; group 2, light grey circles, representing the clade on the bottom right; group 3, dark grey circles, representing the clade on the bottom left). Two of them are relatively distant from each other and even more so from the third clade where most species cluster together. This separation does not correspond with the separation observed based on the whole genome phylogenomic analyses. Only some of the enzymes that cluster together belong to species from the same phylogenomic group. This indicates that the evolution of these enzymes does not correspond to the evolution of the *Streptomyces* species, and that a different force is driving how these enzymes evolved.

### Phylogenetic analysis of *epi*-isozizaene synthases

**Figure 5:**
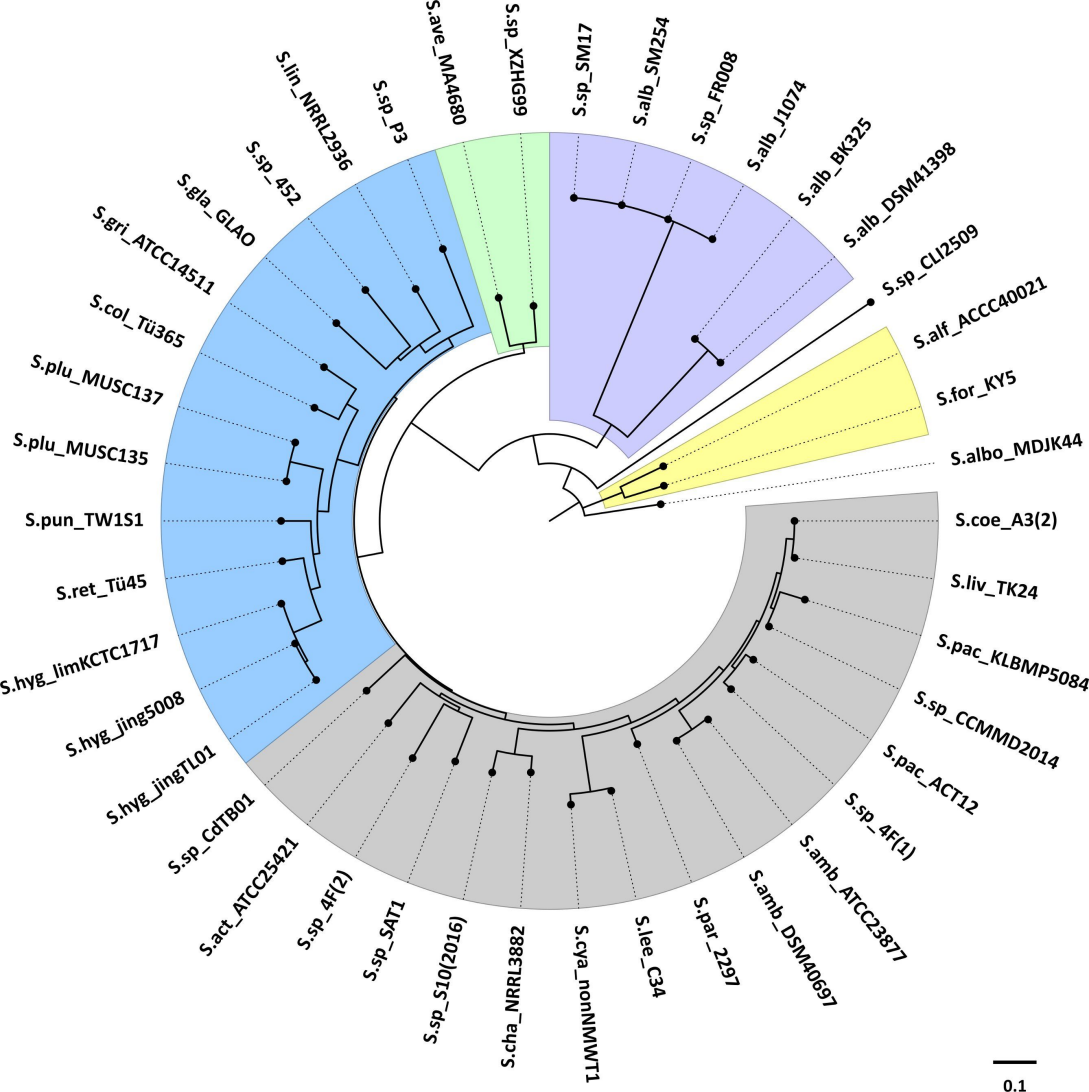
Phylogenetic tree of *epi*-isozizaene synthases. Unrooted maximum likelihood phylogenetic tree of 42 *epi*-isozizaene synthases from the *Streptomyces* species present in the phylogenomic tree in Figure 1. The GenBank accession numbers of the *epi*-isozizaene synthases are listed in Table S5 (Supporting Information File 1).

*epi*-Isozizaene synthases are terpene synthases belonging only to a specific phylogenomic group of *Streptomyces* species. The phylogenetic analysis presented in Figure 5 shows two clades containing most of *epi*-isozizaene synthases and three other minor clades. Not all the enzymes are clustering in the same way as their containing species based on the whole-genome phylogenetic analyses. For example, *S. pactum* ACT12 *epi*-isozizaene synthase clusters together with that of *Streptomyces* sp. 4F, while these two species were present in different branches in the full-genome-based phylogenomic tree. *Streptomyces* sp. 4F clustered together with *S. qaidamensis* S10(2016) and *S. chartreusis* NRRL 3882 in the phylogenomic tree. However, a second *epi*-isozizaene synthase present in *Streptomyces* sp. 4F clusters together with that of *Streptomyces* sp. SAT1, while these two species were located in separate clades of the phylogenomic tree. The occurrence of two genes for terpene synthases with putatively the same function may more strongly point to horizontal gene transfer events. Other cases include the *epi*-isozizaene synthases from *Streptomyces* sp. 452, *S. glaucescens* GLAO, *S. lincolnensis* NRRL 2936 and *Streptomyces* sp. P3 that group together with other enzymes, different to those belonging to species located in their same clade in the whole-genome phylogenomic analyses. This indicates also that some of these terpene synthases have evolved independently of the evolution of the *Streptomyces* species.

### Phylogeny of terpene synthases does not correspond to species-level taxonomy

The comparison of the *Streptomyces* species whole genome-based phylogenetic tree and the three terpene synthase trees shows that not all three comparing phylogenies are congruent. All *Streptomyces* strains included in this study carry at least one copy of *geoA*, with one exception. However, the topology of the geosmin synthase tree is not in harmony with the species tree and only some tips of the trees are conserved (Figure S3, Supporting Information File 1). The topological incongruence is even higher for *epi*-isozizaene and 2-MIB synthase trees (Figures S4 and S5, Supporting Information File 1). Tree reconstruction artefacts cannot explain these incongruences because all phylogenies obtained good statistical support. These data support horizontal gene transfers of terpene synthase genes in *Streptomyces*, but could also point to secondary metabolite genes as being less conserved than housekeeping (primary metabolism) genes. Rapid evolution of secondary metabolism can lead to new natural products with advanced ecological functions in specific ecological niches. If horizontal gene transfer is indeed of high importance, one intriguing question would be why there are almost no *Streptomyces* strains with two or more genes for geosmin synthases and *epi*-isozizaene synthases. This could be explained by the rapid loss of genetic information after uptake of redundant information. It may also reflect the mechanism of integration of the incoming genetic information into the chromosome of the target organism by homologous recombination within identical or highly similar nucleotide sequences. In this study, we searched for the minimal number of events that are required to reconcile the terpene synthase trees with the species tree by performing NOTUNG analyses [52] (for detailed explanations cf. Supporting Information File 1, pp. 33–35). The analyses indicated that the discrepancies between the terpene synthase trees and the species tree, can be explained by horizontal gene transfer of the genes encoding for terpene synthases.

## Conclusion

Overall, this study confirmed that *Streptomyces* species divide in three phylogenomic groups, based here on their whole genomes. Analysis of the distribution of the ten most abundant classes of terpene synthases in *Streptomyces* led to the surprising result that some terpene synthases are restricted to one phylogenomic group or even a subgroup which may point to a specific ecological function of the terpene for the respective group of organisms. The phylogenetic analyses of terpene synthases are not congruent with the phylogenomic analyses. Hence, the evolution of these enzymes does not correspond to the evolution of the *Streptomyces* species, possibly pointing to horizontal gene transfers as an important mechanism involved in the distribution of terpene synthase genes.

In this study, we focused on the distribution and evolution of terpenes synthases among *Streptomyces* species. It would be interesting in follow-up studies to assess the distribution and evolution of these genes among other bacteria, fungi, protists and plants. In addition, a deeper knowledge of the ecological function of terpenes in bacteria and in the interaction with their environment is highly desired.

## Experimental

### *Streptomyces* genomes selection

Genomes with whole sequences available in the NCBI database (thus not partial sequences) were included. Custom shell scripts (https://github.com/kumarsaurabh20/distribution_of_terpene_synthases) were used to filter and download the nucleotide and protein sequences of all complete genomes including an annotation file in GFF format. The 93 selected sequences and their accession numbers are listed in Table S2 (Supporting Information File 1).

### Construction of orthologous gene families

Sequence data of the proteins from the 93 *Streptomyces* species described above were collected. After removing sequences shorter than 50 amino acids, a total of 171,033 sequences were used to construct orthologous gene families using OrthoFinder – v 2.2.6 [53] applying the default setting (BLASTp e-value cut-off = 1e−5; MCL inflation I = 1.5). Using single-copy orthologues a species tree was inferred from unrooted gene trees that were constructed from all single copy genes using the STAG algorithm and the species tree was rooted using the STRIDE algorithm [54]. Both tools are available as core utilities in the OrthoFinder pipeline.

### Phylogenetic analyses

Phylogenetic analyses on three different terpene synthases (geosmin synthases, 2-MIB synthases and *epi*-isozizaene synthases) were performed. Protein and nucleotide sequences were extracted from the *Streptomyces* genomes based on their distribution. Phylogenetic trees were generated using the protein dataset. Sequences were aligned with Mafft version 7.313 [55] using default parameters including *--auto* and *--inputorder*. All the alignments were trimmed for gaps and ambiguously aligned regions with BMGE – v 1.12 [56] using default parameters. For phylogenetic analyses, ProtTest – v 3.1.2 [57] was used to evaluate all evolutionary models under a AIC and BIC criterion. Maximum likelihood analyses were performed in RAxML – v 8.2.12 [58] under JTT+I+G (PROTGAMAMALG) model with rapid bootstrapping of 1000 replicates. GenBank accessions for each sequence are shown in Tables S3 to S5 in Supporting Information File 1.

### Molecular evolution analysis

The coding DNA sequence (CDS) of the three terpene synthase genes (coding for geosmin synthases, 2-MIB synthases and *epi*-isozizaene synthases) in the 93 *Streptomyces* species were collected and aligned with Mafft version 7.313 using default parameters. Geneious – v 9.1 [59] was used to correct frame shifts and premature stop codons. Scripts published in [60] were used to generate codon-based alignments. We used HyPhy instance [61] to perform molecular evolution analysis. To test if positive selection occurred on a proportion of branches in the terpene synthase trees, the SLAC [62] model was used which is an improved version of the commonly used branch-site model. To test the hypothesis that individual sites have been subjected to episodic, positive or diversifying selection, site-specific model FUBAR [63] was used. Additionally, aBSREL [64] model was used to infer nonsynonymous (dN) and synonymous (dS) substitution rates on a per-site basis for a given coding sequence alignment and corresponding phylogeny. The treefix-DTL (duplication-transfer-loss) software, version 1.0.2 [64], was applied to fix the topology of each terpene synthase tree under default settings with an alpha value of 0.05 for the paired-site test and the model closest to PROTGAMMALGF available via treefix-DTL (PROTGAMMAJTTF) as RAxML substitution model. To reconstruct the types and numbers of the evolutionary events that explain the discrepancies (if any) between the final topologies, NOTUNG version 2.9 [52] was run under default settings (modified weight parameters edge weight = 0.9; duplication weight = 2.0; transfer weight = 3.0; losses weight = 1.0) except for the permission of horizontal transfers and the use of a DTL cost matrix of 2-3-1, corresponding to default costs used by treefix-DTL.

## Supporting Information

File 1 Additional figures and tables.
