# Supplementary material for: Phylogenomic analyses and distribution of terpene synthases among Streptomyces
Source: Beilstein J Org Chem. 2019 May 29;15:1181–93. doi: 10.3762/bjoc.15.115 (PMC6604706; doi:10.3762/bjoc.15.115)
Supplement: File 1 — Additional figures and tables. [file Beilstein_J_Org_Chem-15-1181-s001.pdf]

BEILSTEIN JOURNAL OF ORGANIC CHEMISTRY

Supporting Information

for

Phylogenomic analyses and distribution of terpene synthases among *Streptomyces*

Lara Martín-Sánchez, Kumar Saurabh Singh, Mariana Avalos, Gilles P. van Wezel, Jeroen S. Dickschat and Paolina Garbeva

Beilstein J. Org. Chem. **2019,** *15,* 1181–1193. doi:10.3762/bjoc.15.115

Additional figures and tables

License and Terms: This is a supporting information file under the terms of the Creative Commons Attribution License (http://creativecommons.org/licenses/by/4.0). Please note that the reuse, redistribution and reproduction in particular requires that the authors and source are credited.
The license is subject to the *Beilstein Journal of Organic Chemistry* terms and conditions: (https://www.beilstein-journals.org/bjoc)

Table of content

Figures	
S1	Whole-genome phylogenetic analyses of *Streptomyces* species with outgroups
S2	Distribution of dN-dS over individual sites
S3	DTL analyses of geosmin synthases
S4	DTL analyses of 2-methylisoborneol synthases
S5	DTL analyses of *epi*-isozizaene synthases
S6	DTL analyses of geosmin synthases
S7	DTL analyses of 2-methylisoborneol synthases
S8	DTL analyses of epi-isozizaene synthases
**Tables**	
S1	Summary on orthologue analysis based on 93 *Streptomyces* genomes using OrthoFinder
S2	*Streptomyces* genomes used for constructing the phylogenetic trees in Figure 1 and Supplementary Figure S1
S3	List of geosmin synthases used to build the phylogenetic tree in Figure 3
S4	List of 2-methylisoborneol synthases used to build the phylogenetic tree in Figure 4
S5	List of *epi*-isozizaene synthases used to build the phylogenetic tree in Figure 5
S6	List of geosmin synthases used to build the DTL tree in Figure S2
S7	List of 2-methylisoborneol synthases used to build the DTL tree in Figure S3
S8	List of *epi*-isozizaene synthases used to build the DTL tree in Figure S4
S9	Habitats of the *Streptomyces* species represented in the whole genome-based phylogenetic tree

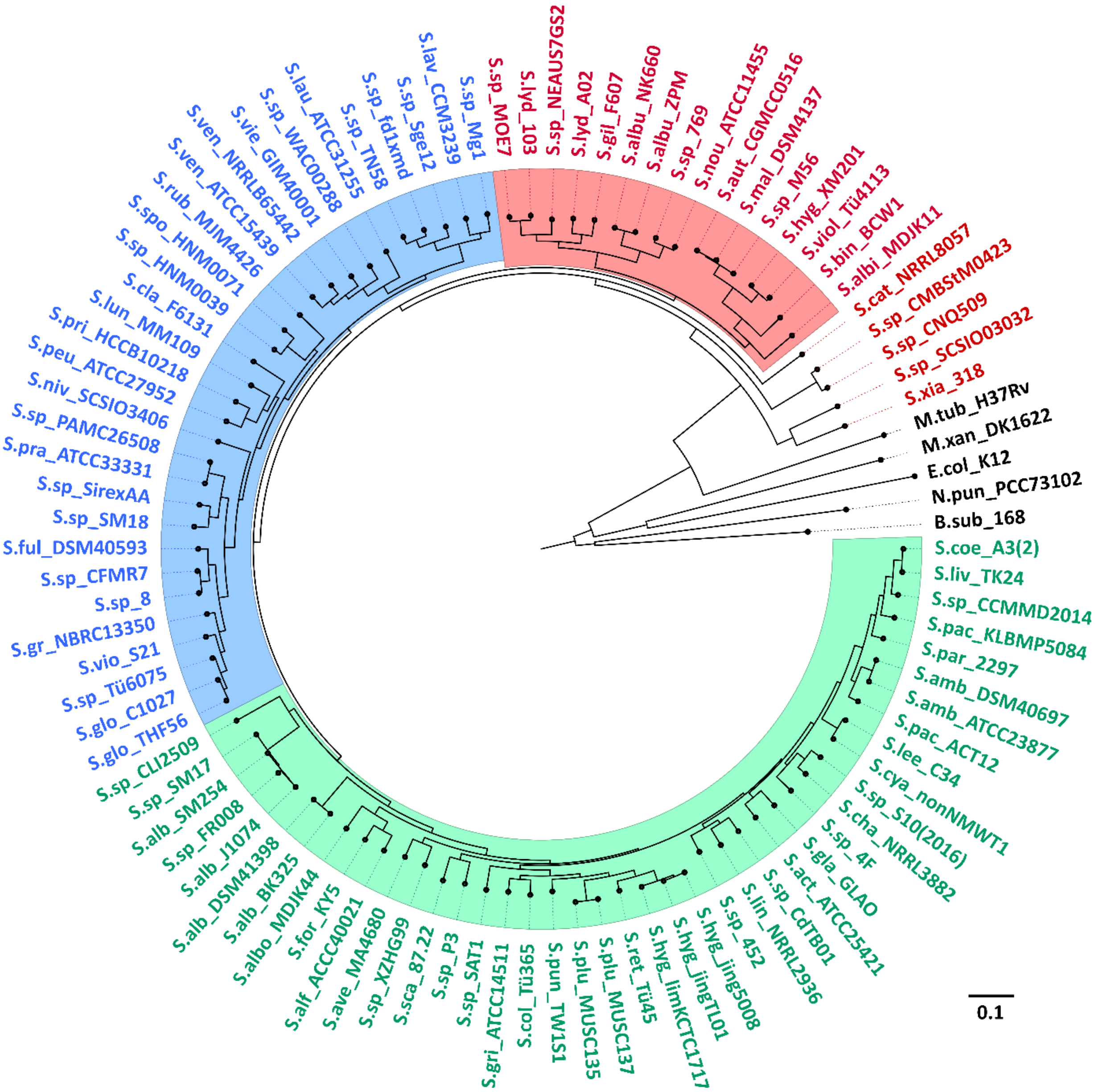

Figure S1. Whole-genome phylogenetic analyses of *Streptomyces* species with outgroups. Rooted maximum likelihood phylogeny of 93 *Streptomyces* species and 5 non-*Streptomyces* species as outgroups, with fully sequenced genomes based on 575 conserved single copy orthologues. All the tools from multiple sequence alignment to consensus tree generation were implemented in OrthoFinder [1]. The species separated in three main groups indicated by different colour-shaded areas. The GenBank accession numbers of the sequences are provided in Supplementary Table S2.

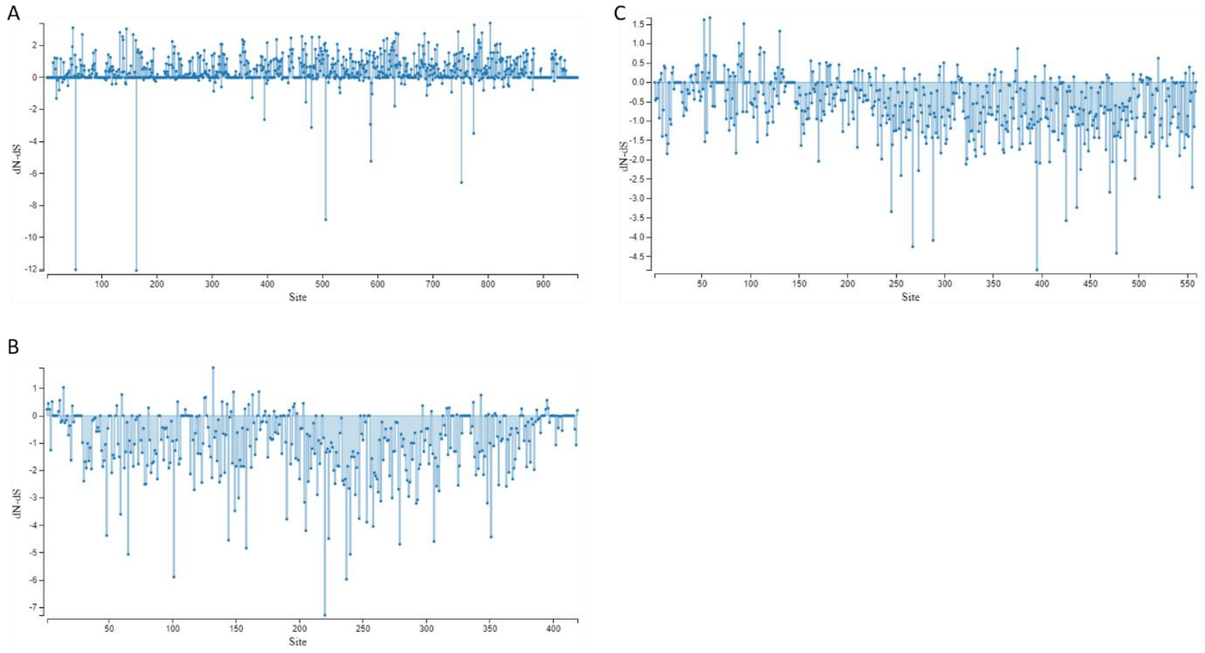

Figure S2. Distribution of dN-dS over individual sites, based on SLAC, for A) geosmin synthase B) *epi*-isozizaene and C) 2-methylisoborneol synthase.

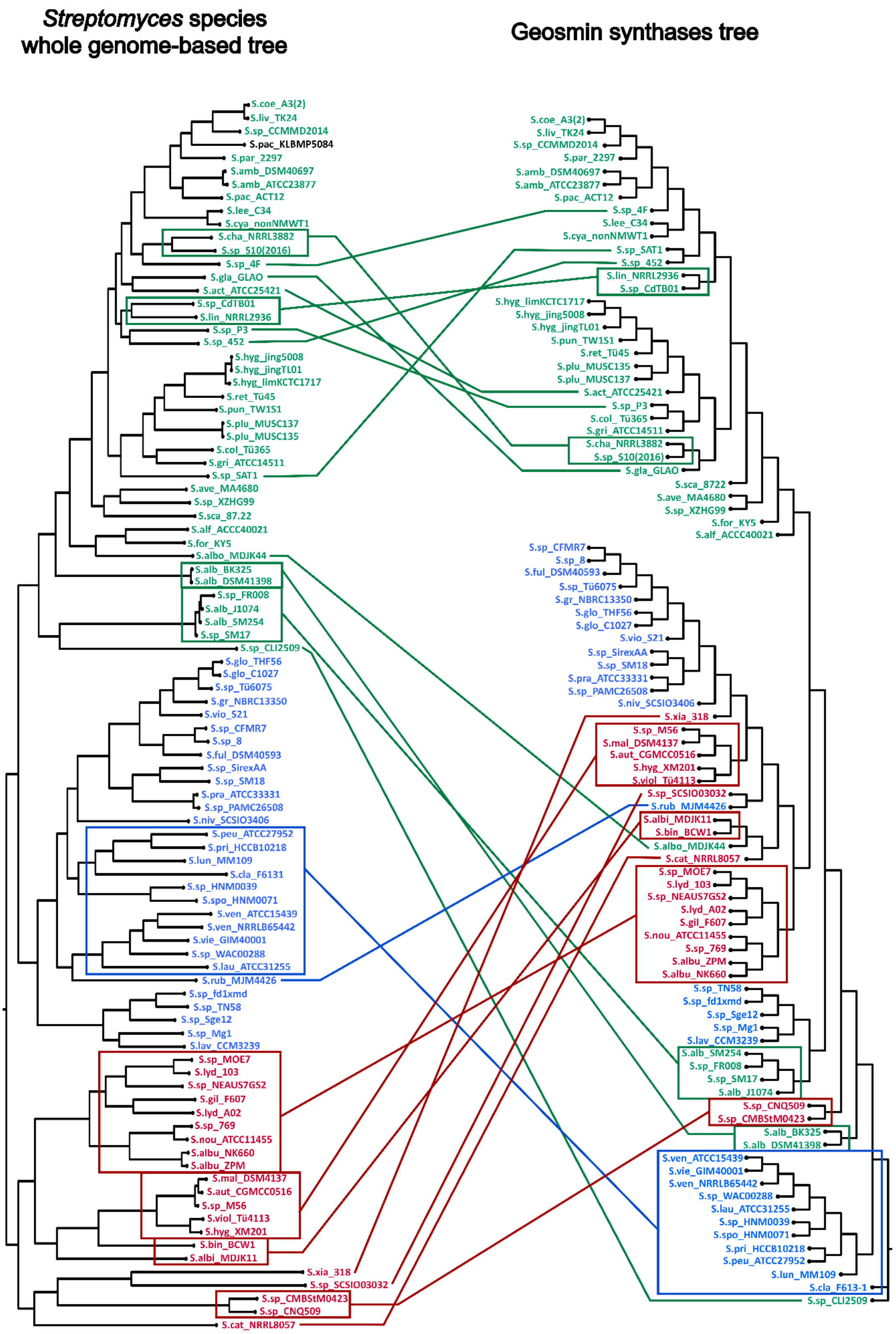

Figure S3. Comparison of the *Streptomyces* species whole genome-based phylogenetic tree with the phylogenetic tree of the geosmin synthases. Confronted linearised versions of the phylogenetic trees in Figure 1 (*Streptomyces* species genomes) and Figure 3 (geosmin synthases). The only species not containing a geosmin synthase is shown in black on the species tree on the left. Discrepancies between both trees are indicated with connecting lines.

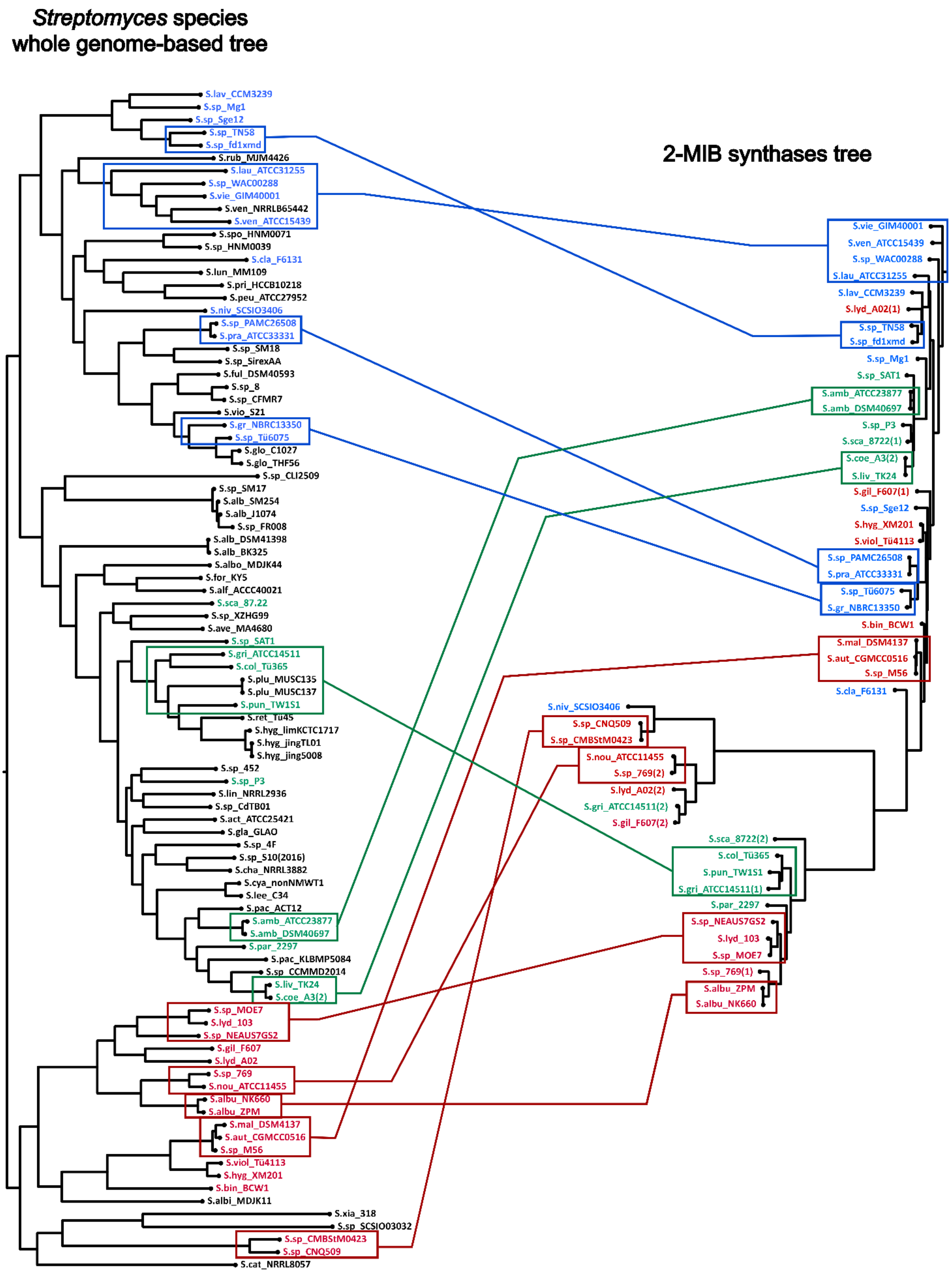

Figure S4. Comparison of the *Streptomyces* species whole genome-based phylogenetic tree with the phylogenetic tree of the 2-methylisoborneol (2-MIB) synthases. Confronted linearised versions of the phylogenetic trees in Figure 1 (*Streptomyces* species genomes) and Figure 4 (2-MIB synthases). The species that do not contain a 2-MIB synthase are shown in black on the species tree on the left. Most of the 2-MIB synthases show discrepancies compared to the species tree. Examples of discrepancies between groups of 2-MIB synthases are shown with connecting lines.

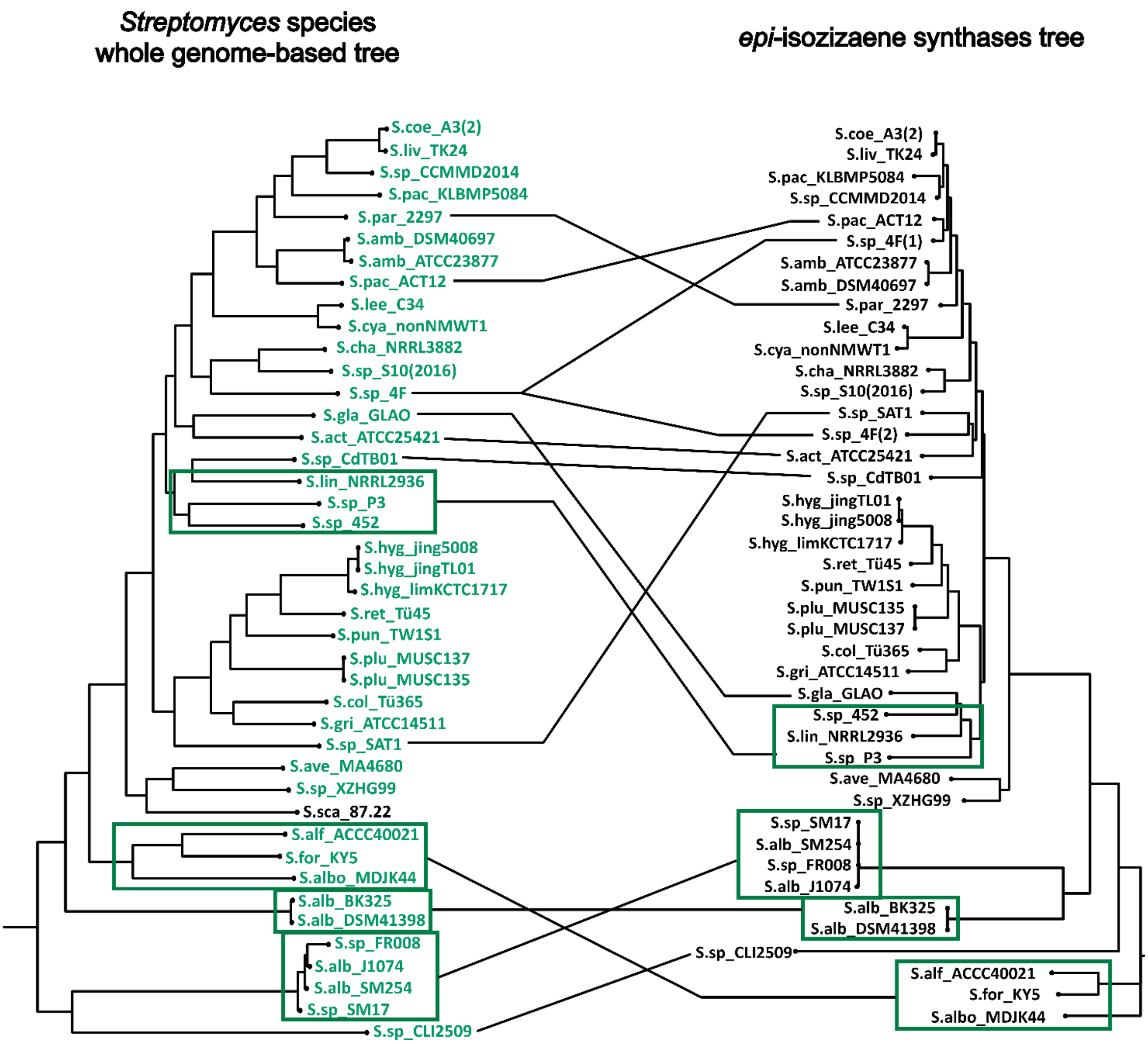

Figure S5. Comparison of the *Streptomyces* species whole genome-based phylogenetic tree with the phylogenetic tree of the *epi*-isozizaene synthases. Confronted linearised versions of the phylogenetic trees in Figure 1 (*Streptomyces* species genomes) and Figure 5 (*epi*-isozizaene synthases). Only one of the clades of the species tree is shown (indicated as the green clade in Figure 1); none of the species from the other two clades harbour an *epi*-isozizaene synthase. The only species not containing an *epi*-isozizaene synthase is shown in black on the species tree on the left. Discrepancies between both trees are indicated with connecting lines.

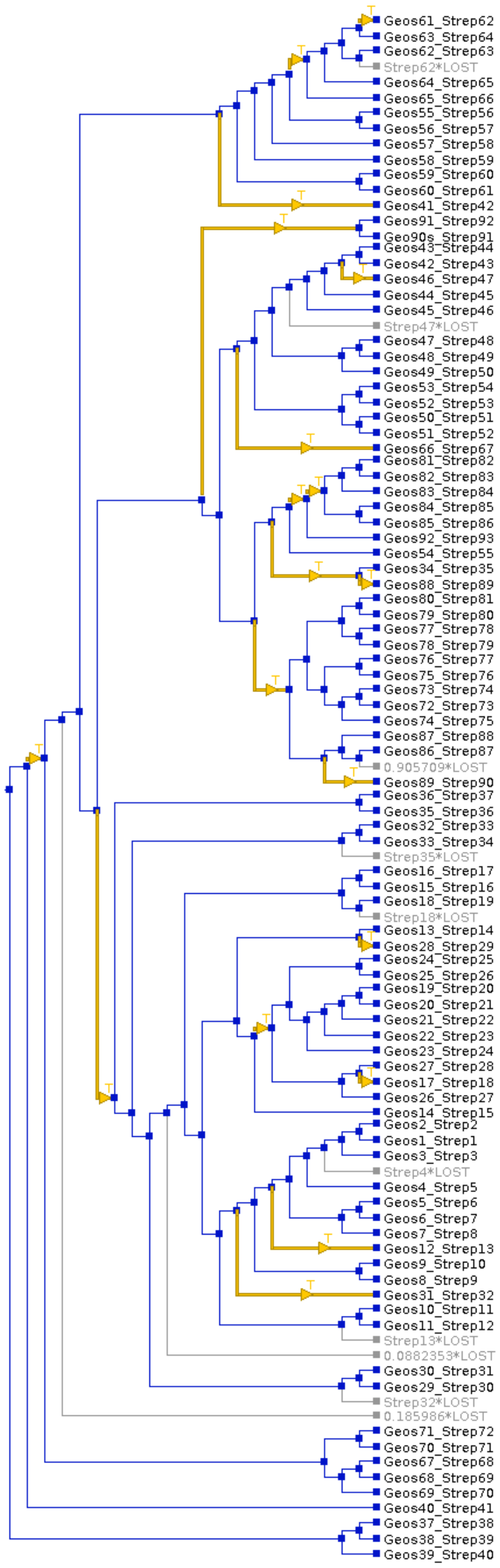

Image generated with Notung 2.9, on Feb 19, 2019

Figure S6. DTL analyses of geosmin synthases. T, Transfer node. Blue square, Speciation node. Node labels in grey indicate that there was gene loss and node labels in black (bold) indicate that there was congruence between the enzyme tree and the species tree. The names on the node labels refer to a particular enzyme and the species harbouring it (see Table S6).

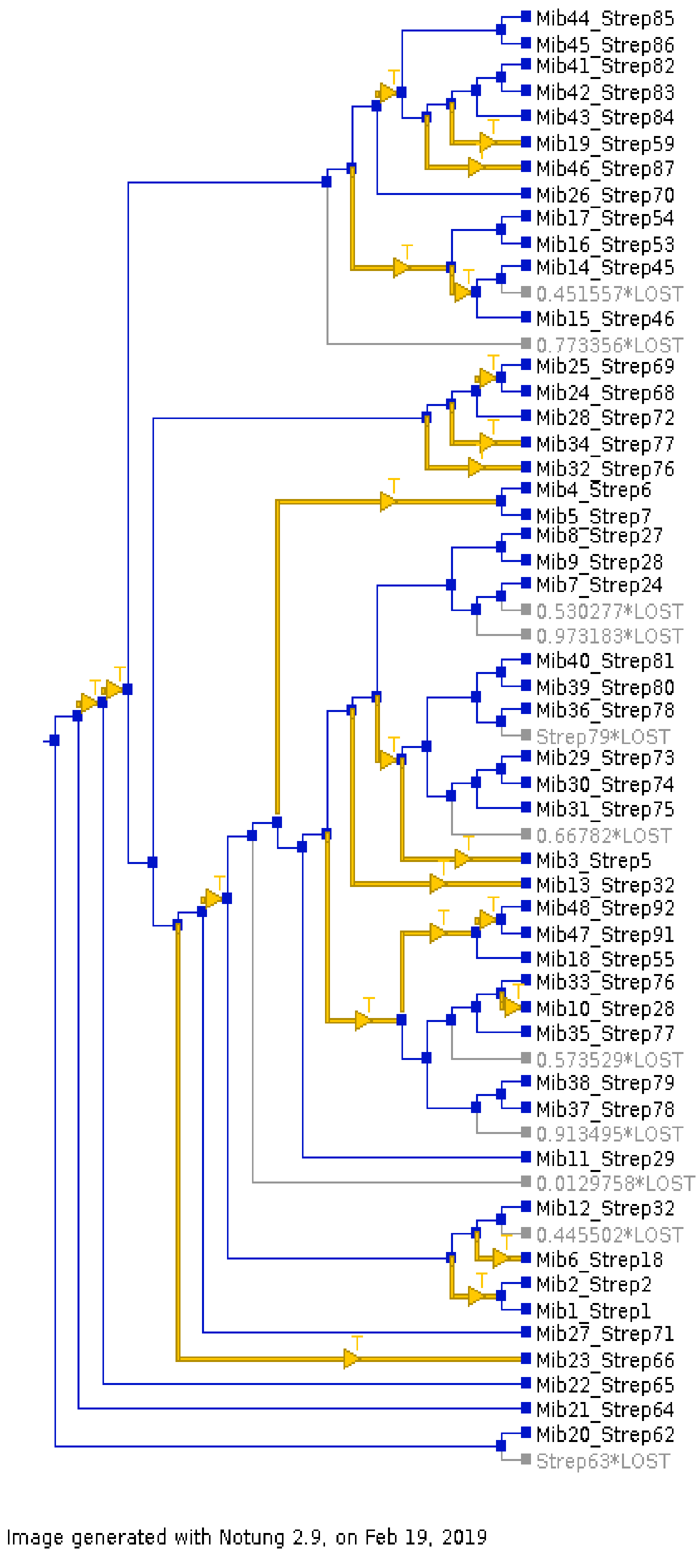

Figure S7. DTL analyses of 2-methylisoborneol synthases. T, Transfer node. Blue square, Speciation node. Node labels in grey indicate that there was gene loss and node labels in black (bold) indicate that there was congruence between the enzyme tree and the species tree. The names on the node labels refer to a particular enzyme and the species harbouring it (see Table S7).

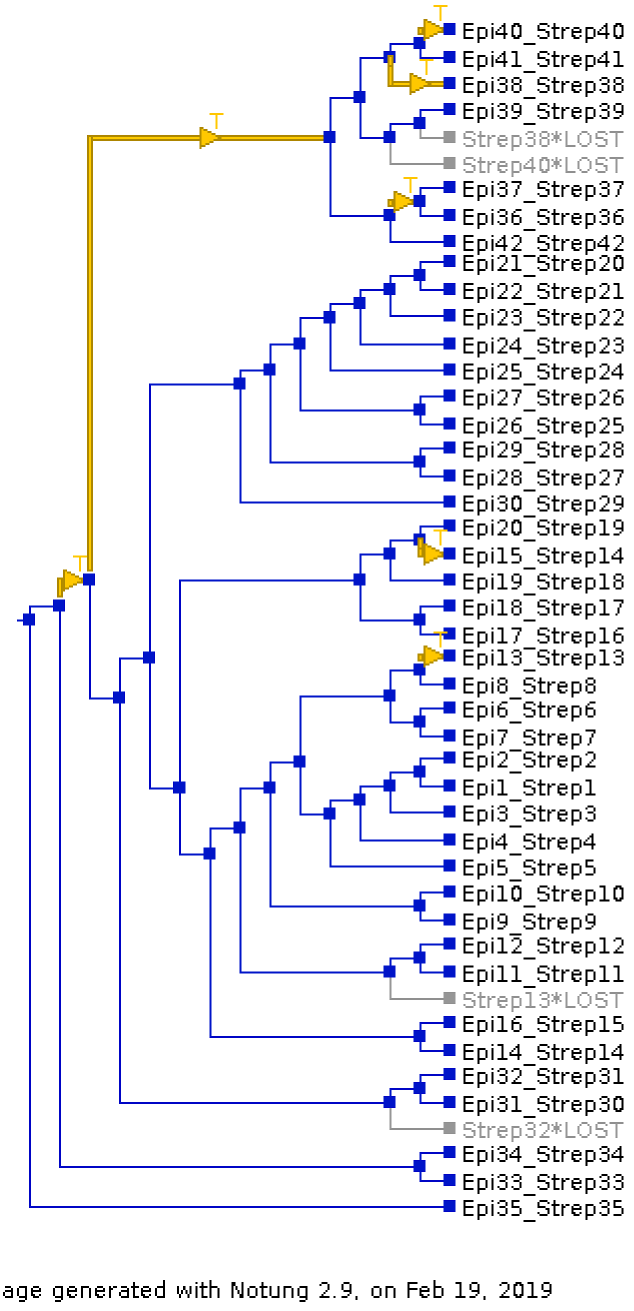

Figure S8. DTL analyses of *epi*-isozizaene synthases. T, Transfer node. Blue square, Speciation node. Node labels in grey indicate that there was gene loss and node labels in black (bold) indicate that there was congruence between the enzyme tree and the species tree. The names on the node labels refer to a particular enzyme and the species harbouring it (see Table S8).

Table S1: Summary on orthologue analysis based on 93 *Streptomyces* genomes using OrthoFinder.

Properties	Value
Number of genes	676554
Number of genes in orthogroups	659764
Number of unassigned genes	16829
Percentage of genes in orthogroups	97.5
Percentage of unassigned genes	2.5
Number of orthogroups (gene families)	19980
Number of species-specific orthogroups	100
Number of genes in species-specific orthogroups	230
Percentage of genes in species-specific orthogroups	0.0
Mean orthogroup size	33
Median orthogroup size	8.0
G50 (assigned genes)	93
G50 (all genes)	93
O50 (assigned genes)	2400
O50 (all genes)	2491
Number of orthogroups with all species present	1156
Number of single-copy orthogroups	575

Table S2: *Streptomyces* genomes used for constructing the phylogenetic trees in Figure 1 and Figure S1.

Abbreviation	Accession number	Species name
S.act_ATCC25421	GCA_003208035.1	*S. actuosus* ATCC 25421
S.alb_J1074	GCA_000359525.1	*S. albidoflavus* J1074
S.albi_MDJK11	GCA_002192455.1	*S. albireticuli* MDJK11
S.albo_MDJK44	GCA_002189675.2	*S. alboflavus* MDJK44
S.albu_NK660	GCA_000695235.1	*S. albulus* NK660
S.albu_ZPM	GCA_000963515.1	*S. albulus* ZPM
S.alb_BK325	GCA_001753425.1	*S. albus* BK3-25
S.alb_DSM41398	GCA_000827005.1	*S. albus* DSM 41398
S.alb_SM254	GCA_001577385.1	*S. albus* SM254
S.alf_ACCC40021	GCA_001975025.1	*S. alfalfae* ACCC40021
S.amb_ATCC23877	GCA_001267885.1	*S. ambofaciens* ATCC 23877
S.amb_DSM40697	GCA_001632865.1	*S. ambofaciens* DSM 40697
S.aut_CGMCC0516	GCA_001983975.1	*S. autolyticus* CGMCC0516
S.ave_MA4680	GCA_000009765.2	*S. avermitilis* MA-4680
S.bin_BCW1	GCA_000092385.1	*S. bingchenggensis* BCW-1
S.cat_NRRL8057	GCA_000237305.1	*S. cattleya* NRRL 8057 = DSM 46488
S.cha_NRRL3882	GCA_900236475.1	*S. chartreusis* NRRL 3882
S.cla_F6131	GCA_001693675.1	*S. clavuligerus* F613-1
S.coe_A3(2)	GCA_000203835.1	*S. coelicolor* A3(2)
S.col_Tü365	GCA_000444875.1	*S. collinus* Tü 365
S.cya_nonNMWT1	GCA_000931445.1	*S. cyaneogriseus subsp. noncyanogenus* NMWT 1
S.for_KY5	GCA_002556545.1	*S. formicae* KY5
S.ful_DSM40593	GCA_000385945.1	*S. fulvissimus* DSM 40593
S.gil_F607	GCA_002082195.1	*S. gilvosporeus* F607
S.gla_GLAO	GCA_000761215.1	*S. glaucescens* GLA.O
S.glo_C1027	GCA_000261345.2	*S. globisporus* C-1027
S.glo_THF56	GCA_003147545.1	*S. globisporus* THF56
S.gri_ATCC14511	GCA_001542625.2	*S. griseochromogenes* ATCC 14511
S.gr_NBRC13350	GCA_000010605.1	*S. griseus subsp. griseus* NBRC 13350

S.hyg_jing5008	GCA_000245355.1	*S. hygroscopicus subsp. jinggangensis* 5008
S.hyg_jingTL01	GCA_000340845.1	*S. hygroscopicus subsp. jinggangensis* TL01
S.hyg_limKCTC1717	GCA_001447075.1	*S. hygroscopicus subsp. limoneus* KCTC 1717
S.hyg_XM201	GCA_002021875.1	*S. hygroscopicus* XM201
S.lau_ATCC31255	GCA_002355495.1	*S. laurentii* ATCC 31255
S.lav_CCM3239	GCA_002803845.1	*S. lavendulae subsp. lavendulae* CCM 3239
S.lee_C34	GCA_001013905.1	*S. leeuwenhoekii* C34
S.lin_NRRL2936	GCA_001685355.1	*S. lincolnensis* NRRL 2936
S.liv_TK24	GCA_000739105.1	*S. lividans* TK24
S.lun_MM109	GCA_003054555.1	*S. lunaelactis* MM109
S.lyd_103	GCA_001729485.1	*S. lydicus* 103
S.lyd_A02	GCA_000952035.2	*S. lydicus* A02
S.mal_DSM4137	GCA_002591335.1	*S. malaysiensis* DSM 4137
S.niv_SCSIO3406	GCA_002009175.1	*S. niveus* SCSIO 3406
S.nou_ATCC11455	GCA_001704275.1	*S. noursei* ATCC 11455
S.pac_ACT12	GCA_002005225.1	*S. pactum* ACT12
S.pac_KLBMP5084	GCA_001767375.1	*S. pactum* KLBMP 5084
S.par_2297	GCA_001660045.1	*S. parvulus* 2297
S.peu_ATCC27952	GCA_002777535.1	*S. peucetius subsp. caesius* ATCC 27952
S.plu_MUSC135	GCA_000802245.2	*S. pluripotens* MUSC 135
S.plu_MUSC137	GCA_000816465.4	*S. pluripotens* MUSC 137
S.pra_ATCC33331	GCA_000176115.2	*S. pratensis* ATCC 33331
S.pri_HCCB10218	GCA_001278075.1	*S. pristinaespiralis* HCCB 10218
S.pun_TW1S1	GCA_001735805.1	*S. puniciscabiei* TW1S1
S.ret_Tü45	GCA_001511815.1	*S. reticuli* Tü 45
S.rub_MJM4426	GCA_001750785.1	*S. rubrolavendulae* MJM4426
S.sca_87.22	GCA_000091305.1	*S. scabiei* 87.22
S.sp_452	GCA_003074055.1	*S. sp.* 452 (*S. nigra* 452)
S.sp_4F	GCA_001484705.1	*S. sp.* 4F
S.sp_769	GCA_000816025.1	*S. sp.* 769
S.sp_CCMMD2014	GCA_000772045.1	*S. sp.* CCM_MD2014
S.sp_CdTB01	GCA_001484565.1	*S. sp.* CdTB01

S.sp_CFMR7	GCA_001278095.1	*S. sp.* CFMR 7
S.sp_CLI2509	GCA_002288075.1	*S. sp.* CLI2509
S.sp_CMBStM0423	GCA_002847285.1	*S. sp.* CMB-StM0423
S.sp_CNQ509	GCA_001011035.1	*S. sp.* CNQ-509
S.sp_fd1xmd	GCA_002007685.1	*S. sp.* fd1-xmd
S.sp_FR008	GCA_001431765.1	*S. sp.* FR-008
S.sp_HNM0039	GCA_003097515.1	*S. sp.* HNM0039
S.sp_M56	GCA_002812405.1	*S. sp.* M56
S.sp_Mg1	GCA_000412265.2	*S. sp.* Mg1
S.sp_MOE7	GCA_002090335.1	*S. sp.* MOE7
S.sp_NEAUS7GS2	GCA_003173275.1	*S. sp.* NEAU-S7GS2
S.sp_P3	GCA_003032475.1	*S. sp.* P3
S.sp_PAMC26508	GCA_000364805.1	*S. sp.* PAMC 26508
S.sp_S10(2016)	GCA_001611795.1	*S. sp.* S10(2016) (*S. qaidamensis* S10(2016))
S.sp_8	GCA_002094995.1	*S. sp.* S8
S.sp_SAT1	GCA_001654495.1	*S. sp.* SAT1
S.sp_SCSIO03032	GCA_002128305.1	*S. sp.* SCSIO 03032
S.sp_Sge12	GCA_002080455.1	*S. sp.* Sge12
S.sp_SirexAA	GCA_000177195.2	*S. sp.* SirexAA-E
S.sp_SM17	GCA_002910725.2	*S. sp.* SM17
S.sp_SM18	GCA_002910775.2	*S. sp.* SM18
S.sp_TN58	GCA_001941845.1	*S. sp.* TN58
S.sp_Tü6075	GCA_001931635.1	*S. sp.* Tü 6075
S.sp_WAC00288	GCA_002943895.1	*S. sp.* WAC00288
S.sp_XZHG99	GCA_002946835.1	*S. sp.* XZHG99 (*S. dengpaensis* XZHG99)
S.spo_HNM0071	GCA_003122365.1	*S. spongiicola* HNM0071
S.ven_ATCC15439	GCA_001443625.1	*S. venezuelae* ATCC 15439
S.ven_NRRLB65442	GCA_001886595.1	*S. venezuelae* NRRL B-65442
S.vie_GIM40001	GCA_000830005.1	*S. vietnamensis* GIM4.0001
S.vio_S21	GCA_002082175.1	*S. violaceoruber* S21
S.viol_Tü4113	GCA_000147815.3	*S. violaceusniger* Tü 4113
S.xia_318	GCA_000993785.2	*S. xiamenensis* 318

**Outgroups**		
B.sub_168	GCF_000009045.1	*Bacillus subtilis subsp. subtilis* strain 168
M.tub_H37Rv	GCF_000195955.2	*Mycobacterium tuberculosis* H37Rv
E.col_K12	GCF_000005845.2	*Escherichia coli* K12 substr. MG1655
N.pun_PCC73102	GCF_000020025.1	*Nostoc punctiforme* PCC 73102
M.xan_DK1622	GCF_000012685.1	*Myxococcus xanthus* DK1622

Table S3: List of geosmin synthases used to build the phylogenetic tree in Figure 3.

Abbreviation	Accession number	Species name	amino acids
S.act_ATCC25421	WP_110632077	*S. actuosus* ATCC 25421	719
S.alb_J1074	WP_003951048	*S. albidoflavus* J1074	724
S.albi_MDJK11	WP_087929697	*S. albireticuli* MDJK11	758
S.albo_MDJK44	WP_087886527	*S. alboflavus* MDJK44	738
S.albu_NK660	WP_038526177	*S. albulus* NK660	746
S.albu_ZPM	WP_037632171	*S. albulus* ZPM	746
S.alb_BK325	WP_040246537	*S. albus* BK3-25	737
S.alb_DSM41398	WP_040246537	*S. albus* DSM 41398	737
S.alb_SM254	WP_079055460	*S. albus* SM254	724
S.alf_ACCC40021	WP_076683988	*S. alfalfae* ACCC40021	721
S.amb_ATCC23877	WP_053138925	*S. ambofaciens* ATCC 23877	726
S.amb_DSM40697	WP_063483426	*S. ambofaciens* DSM 40697	726
S.aut_CGMCC0516	WP_079258182	*S. autolyticus* CGMCC0516	787
S.ave_MA4680	WP_010983603	*S. avermitilis* MA-4680	725
S.bin_BCW1	WP_014174668	*S. bingchenggensis* BCW-1	751
S.cat_NRRL8057	WP_014143690	*S. cattleya* NRRL 8057	741
S.cha_NRRL3882	WP_010034221	*S. chartreusis* NRRL 3882	720
S.cla_F613-1	ANW21593	*S. clavuligerus* F613-1	724
S.coe_A3(2)	WP_011030632	*S. coelicolor* A3(2)	726
S.col_ Tü365	WP_020942918	*S. collinus* Tü 365	720
S.cya_nonNMWT1	WP_044385074	*S. cyaneogriseus subsp. noncyanogenus* NMWT 1	735
S.for_KY5	WP_098245470	*S. formicae* KY5	721
S.ful_DSM40593	WP_015606689	*S. fulvissimus* DSM 40593	737
S.gil_F607	WP_083106597	*S. gilvosporeus* F607	749
S.gla_GLAO	WP_043504863	*S. glaucescens* GLA.O	725
S.glo_C1027	WP_058953825	*S. globisporus* C-1027	737
S.glo_THF56	WP_044369842	*S. globisporus* TFH56	737
S.gri_ATCC14511	WP_067302609	*S. griseochromogenes* ATCC 14511	729
S.gr_NBRC13350	WP_012382258	*S. griseus subsp. griseus* NBRC 13350	737

S.hyg_jing5008	WP_014675700	*S. hygroscopicus subsp. jinggangensis* 5008	717
S.hyg_jingTL01	WP_014675700	*S. hygroscopicus subsp. jinggangensis* TL01	717
S.hyg_limKCTC1717	WP_058082416	*S. hygroscopicus subsp. limoneus* KCTC 1717	717
S.hyg_XM201	WP_078647520	*S. hygroscopicus* XM201	767
S.lau_ATCC31255	BAU81127	*S. laurentii* ATCC 31255	732
S.lav_CCM3239	WP_030234522	*S. lavendulae subsp. lavendulae* CCM 3239	745
S.lee_C34	WP_047121662	*S. leeuwenhoekii* C34	736
S.lin_NRRL2936	WP_067441821	*S. lincolnensis* NRRL 2936	716
S.liv_TK24	WP_003972847	*S. lividans* TK24	726
S.lun_MM109	WP_108147039	*S. lunaelactis* MM109	736
S.lyd_103	WP_069572885	*S. lydicus* 103	745
S.lyd_A02	WP_046926865	*S. lydicus* A02	745
S.mal_DSM4137	WP_099016999	*S. malaysiensis* DSM 4137	798
S.niv_SCSIO3406	WP_078076350	*S. niveus* SCSIO 3406	771
S.nou_ATCC11455	WP_079143472	*S. noursei* ATCC 11455	724
S.pac_ACT12	WP_055419631	*S. pactum* ACT12	727
S.par_2297	WP_064730371	*S. parvulus* 2297	732
S.peu_ATCC27952	ABY50951	*S. peucetius subsp. caesius* ATCC 27952	732
S.plu_MUSC135	WP_039648506	*S. pluripotens* MUSC 135	720
S.plu_MUSC137	WP_039648506	*S. pluripotens* MUSC 137	720
S.pra_ATCC33331	WP_014158016	*S. pratensis* ATCC 33331	747
S.pri_HCCB10218	WP_005321403	*S. pristinaespiralis* HCCB 10218	734
S.pun_TW1S1	WP_069778096	*S. puniciscabiei* TW1S1	720
S.ret_Tü45	WP_059253583	*S. reticuli* Tü45	720
S.rub_MJM4426	WP_069975090	*S. rubrolavendulae* MJM4426	753
S.sca_8722	WP_012999852	*S. scabiei* 87.22	735
S.sp_452	WP_108709220	*S. sp.* 452 (*S. nigra* 452)	720
S.sp_4F	WP_058917193	*S. sp.* 4F	718
S.sp_769	WP_039641183	*S. sp.* 769	740
S.sp_CCMMD2014	WP_061441960	*S. sp.* CCM_MD2014	733
S.sp_CdTB01	WP_058922365	*S. sp.* CdTB01	718
S.sp_CFMR7	WP_053562493	*S. sp.* CFMR 7	737

S.sp_CLI2509	WP_095682396	*S. sp.* CLI2509	826
S.sp_CMBStM0423	WP_101425785	*S. sp.* CMB-StM0423	755
S.sp_CNQ509	WP_052770207	*S. sp.* CNQ509	756
S.sp_fd1xmd	WP_078095378	*S. sp.* fd1-xmd	743
S.sp_FR008	WP_075986266	*S. sp.* FR-008	724
S.sp_HNM0039	WP_108908415	*S. sp.* HNM0039	751
S.sp_M56	WP_100806825	*S. sp.* M56	787
S.sp_Mg1	WP_008743400	*S. sp.* Mg1	738
S.sp_MOE7	WP_084772522	*S. sp.* MOE7	745
S.sp_NEAUS7GS2	WP_109891303	*S. sp.* NEAU-S7GS2	744
S.sp_P3	WP_107446227	*S. sp.* P3	723
S.sp_PAMC26508	WP_015575862	*S. sp.* PAMC26508	747
S.sp_S10(2016)	WP_062929741	*S. sp.* S10(2016) (*S. qaidamensis* S10(2016))	722
S.sp_8	WP_084996823	*S. sp.* S8	737
S.sp_SAT1	WP_064535845	*S. sp.* SAT1	727
S.sp_SCSIO03032	WP_086159477	*S. sp.* SCSIO 03032	761
S.sp_Sge12	WP_081522109	*S. sp.* Sge12	738
S.sp_SirexAA	WP_014044184	*S. sp.* SirexAA-E	745
S.sp_SM17	WP_030308315	*S. sp.* SM17	724
S.sp_SM18	WP_103493052	*S. sp.* SM18	745
S.sp_TN58	WP_075971290	*S. sp.* TN58	741
S.sp_Tü6075	WP_075268060	*S. sp.* Tü 6075	737
S.sp_WAC00288	WP_062757268	*S. sp.* WAC00288	731
S.sp_XZHG99	WP_099498889	*S. sp.* XZHG99 (*S. dengpaensis* XZHG99)	717
S.spo_HNM0071	WP_109297326	*S. spongiicola* HNM0071	751
S.ven_ATCC15439	WP_055645174	*S. venezuelae* ATCC 15439	732
S.ven_NRRLB65442	WP_015031476	*S. venezuelae* NRRL B-65442	728
S.vie_GIM40001	WP_041127665	*S. vietnamensis* GIM4.0001	732
S.vio_S21	WP_083191937	*S. violaceoruber* S21	744
S.viol_Tü4113	WP_014061818	*S. violaceusniger* Tü 4113	758
S.xia_318	WP_030731770	*S. xiamenensis* 318	748

Table S4: List of 2-MIB synthases used to build the phylogenetic tree in Figure 4.

Abbreviation	Accession number	Species name	amino acids
S. albu_NK660	WP_038524797	*S. albulus* NK660	352
S. albu_ZPM	WP_020930496	*S. albulus* ZPM	352
S. amb_ATCC23877	WP_053126184	*S. ambofaciens* ATCC 23877	440
S. amb_DSM40697	WP_063481016	*S. ambofaciens* DSM 40697	440
S. aut_CGMCC0516	WP_079256828	*S. autolyticus* CGMCC0516	431
S. bin_BCW1	WP_043488086	*S. bingchenggensis* BCW-1	402
S. cla_F6131	ANW17109	*S. clavuligerus* F613-1	400
S. coe_A3(2)	NP_733742	*S. coelicolor* A3(2)	440
S. col_Tü365	WP_020938197	*S. collinus* Tü 365	350
S. gil_F607(1)	WP_083108965	*S. gilvosporeus* F607	442
S. gil_F607(2)	WP_083103453	*S. gilvosporeus* F607	382
S. gri_ATCC14511(1)	WP_067309437	*S. griseochromogenes* ATCC 14511	388
S. gri_ATCC14511(2)	WP_067310286	*S. griseochromogenes* ATCC 14511	350
S. gr_NBRC13350	WP_012378420	*S. griseus subsp. griseus* NBRC 13350	437
S. hyg_XM201	WP_078645903	*S. hygroscopicus* XM201	442
S. lau_ATCC31255	BAU87358	*S. laurentii* ATCC 31255	441
S. lav_CCM3239	WP_078950304	*S. lavendulae subsp. lavendulae* CCM 3239	436
S. liv_TK24	WP_011031839	*S. lividans* TK24	440
S. lyd_103	WP_069571074	*S. lydicus* 103	352
S. lyd_A02(1)	WP_046924697	*S. lydicus* A02	431
S. lyd_A02(2)	WP_078984193	*S. lydicus* A02	445
S. mal_DSM4137	WP_099012977	*S. malaysiensis* DSM 4137	389
S. niv_SCSIO3406	WP_078079412	*S. niveus* SCSIO 3406	382
S. nou_ATCC11455	WP_079143205	*S. noursei* ATCC 11455	394
S. par_2297	WP_064725957	*S. parvulus* 2297	352
S. pra_ATCC33331	WP_014157663	*S. pratensis* ATCC 33331	439
S. pun_TW1S1	WP_069782778	*S. puniciscabiei* TW1S1	356
S. sca_8722(1)	WP_041668842	*S. scabiei* 87.22	454
S. sca_8722(2)	WP_037726550	*S. scabiei* 87.22	354
S. sp_769(1)	WP_039628838	*S. sp.* 769	400

S. sp_769(2)	WP_078876140	*S. sp.* 769	352
S. sp_CMBStM0423	WP_101423704	*S. sp.* CMB-StM0423	400
S. sp_CNQ509	WP_047016550	*S. sp.* CNQ-509	402
S. sp_fd1xmd	WP_078095811	*S. sp.* fd1-xmd	462
S. sp_M56	WP_100807892	*S. sp.* M56	437
S. sp_Mg1	WP_047960430	*S. sp.* Mg1	426
S. sp_MOE7	WP_084775022	*S. sp.* MOE7	352
S. sp_NEAUS7GS2	WP_109889928	*S. sp.* NEAU-S7GS2	353
S. sp_P3	WP_107448681	*S. sp.* P3	451
S. sp_PAMC26508	WP_015576150	*S. sp.* PAMC 26508	439
S. sp_SAT1	WP_064537133	*S. sp.* SAT1	424
S. sp_Sge12	WP_081521483	*S. sp.* Sge12	455
S. sp_TN58	WP_075971735	*S. sp.* TN58	436
S. sp_Tü6075	WP_075263486	*S. sp.* Tue 6075	457
S. sp_WAC00288	WP_062750724	*S. sp.* WAC00288	415
S. ven_ATCC15439	WP_055645320	*S. venezuelae* ATCC 15439	423
S. vie_GIM40001	WP_041132328	*S. vietnamensis* GIM4.0001	464
S. vio_Tü4113	WP_014058647	*S. violaceusniger* Tü 4113	432

Table S5: List of *epi*-isozizaene synthases used to build the phylogenetic tree in Figure 5.

Abbreviation	Accession number	Species name	amino acids
S.act_ATCC25421	WP_110630412	*S. actuosus* ATCC 25421	365
S.albo_MDJK44	WP_087885793	*S. alboflavus* MDJK44	371
S.alb_BK325	WP_107071290	*S. albus* BK3-25	363
S.alb_DSM41398	WP_107071290	*S. albus* DSM 41398	363
S.alb_J1074	WP_008415715	*S. albidoflavus* J1074	324
S.alb_SM254	WP_030765460	*S. albus* SM254	324
S.alf_ACCC40021	APY90801	*S. alfalfae* ACCC40021	343
S.amb_ATCC23877	WP_079030788	*S. ambofaciens* ATCC 23877	363
S.amb_DSM40697	WP_079155896	*S. ambofaciens* DSM 40697	363
S.ave_MA4680	WP_107083301	*S. avermitilis* MA-4680	361
S.cha_NRRL3882	WP_029181723	*S. chartreusis* NRRL 3882	361
S.coe_A3(2)	NP_629369	*S. coelicolor* A3(2)	361
S.col_Tü365	AGS71733.1	*S. collinus* Tü365	337
S.cya_nonNMWT1	WP_044388300	*S. cyaneogriseus subsp. noncyanogenus* NMWT 1	362
S.for_KY5	WP_098244686	*S. formicae* KY5	370
S.gla_GLAO	WP_099052949	*S. glaucescens* GLA.O	358
S.gri_ATCC14511	WP_099053013	*S. griseochromogenes* ATCC 14511	360
S.hyg_jing5008	WP_086011574	*S. hygroscopicus subsp. jinggangensis* 5008	348
S.hyg_jingTL01	WP_086011574	*S. hygroscopicus subsp. jinggangensis* TL01	348
S.hyg_limKCTC1717	ALO96063	*S. hygroscopicus subsp. limoneus* KCTC 1717	361
S.lee_C34	WP_047122496	*S. leeuwenhoekii* C34	362
S.lin_NRRL2936	WP_107406875	*S. lincolnensis* NRRL 2936	361
S.liv_TK24	AIJ13444	*S. lividans* TK24	337
S.pac_ACT12	WP_079160747	*S. pactum* ACT12	363
S.pac_KLBMP5084	WP_078535684	*S. pactum* KLBMP 5084	361
S.par_2297	WP_064731961	*S. parvulus* 2297	361
S.plu_MUSC135	WP_086083749	*S. pluripotens* MUSC 135	361
S.plu_MUSC137	WP_086083749	*S. pluripotens* MUSC 137	361
S.pun_TW1S1	WP_099055058	*S. puniciscabiei* TW1S1	361

S.ret_Tü45	WP_107118101	*S. reticuli* Tü 45	337
S.sp_452	WP_108710823	*S. sp.* 452 (*S. nigra* 452)	361
S.sp_4F(1)	OSC69340	*S. sp.* 4F	363
S.sp_4F(2)	WP_058917971	*S. sp.* 4F	361
S.sp_CCMMD2014	WP_061446904	*S. sp.* CCM MD2014	361
S.sp_CdTB01	WP_107416269	*S. sp.* CdTB01	361
S.sp_CLI2509	WP_095682130.1	*S. sp.* CLI2509	353
S.sp_FR008	WP_030765460	*S. sp.* FR-008	324
S.sp_P3	WP_107441985	*S. sp.* P3	353
S.sp_S10(2016)	WP_107308381	*S. sp.* S10(2016) (*S. qaidamensis* S10(2016))	350
S.sp_SAT1	WP_107440814	*S. sp.* SAT1	367
S.sp_SM17	AWL34654	*S. sp.* SM17	324
S.sp_XZHG99	WP_099500021	*S. sp.* XZHG99 (*S. dengpaensis* XZHG99)	361

Table S6: List of geosmin synthases used to build the DTL tree in Figure S6.

Abbreviation	Accession number	Species name
Geos1_Strep1	WP_011030632	*S. coelicolor* A3(2)
Geos2_Strep2	WP_003972847	*S. lividans* TK24
Geos3_Strep3	WP_061441960	*S. sp.* CCM_MD2014
Geos4_Strep5	WP_064730371	*S. parvulus* 2297
Geos5_Strep6	WP_063483426	*S. ambofaciens* DSM 40697
Geos6_Strep7	WP_053138925	*S. ambofaciens* ATCC 23877
Geos7_Strep8	WP_055419631	*S. pactum* ACT12
Geos8_Strep9	WP_047121662	*S. leeuwenhoekii* C34
Geos9_Strep10	WP_044385074	*S. cyaneogriseus subsp. noncyanogenus* NMWT 1
Geos10_Strep11	WP_010034221	*S. chartreusis* NRRL 3882
Geos11_Strep12	WP_062929741	*S. sp.* S10(2016)
Geos12_Strep13	WP_058917193	*S. sp.* 4F
Geos13_Strep14	WP_043504863	*S. glaucescens* GLA.O
Geos14_Strep15	WP_110632077	*S. actuosus* ATCC 25421
Geos15_Strep16	WP_058922365	*S. sp.* CdTB01
Geos16_Strep17	WP_067441821	*S. lincolnensis* NRRL 2936
Geos17_Strep18	WP_107446227	*S. sp.* P3
Geos18_Strep19	WP_108709220	*S. sp.* 452
Geos19_Strep20	WP_014675700	*S. hygroscopicus subsp. jinggangensis* 5008
Geos20_Strep21	WP_014675700_1	*S. hygroscopicus subsp. jinggangensis* TL01
Geos21_Strep22	WP_058082416	*S. hygroscopicus subsp. limoneus* KCTC 1717
Geos22_Strep23	WP_059253583	*S. reticuli* Tü45
Geos23_Strep24	WP_069778096	*S. puniciscabiei* TW1S1
Geos24_Strep25	WP_039648506	*S. pluripotens* MUSC 135
Geos25_Strep26	WP_039648506_1	*S. pluripotens* MUSC 137
Geos26_Strep27	WP_020942918	*S. collinus* Tü 365
Geos27_Strep28	WP_067302609	*S. griseochromogenes* ATCC 14511
Geos28_Strep29	WP_064535845	*S. sp.* SAT1
Geos29_Strep30	WP_010983603	*S. avermitilis* MA-4680

Geos30_Strep31	WP_099498889	*S. sp.* XZHG99
Geos31_Strep32	WP_012999852	*S. scabiei* 87.22
Geos32_Strep33	WP_076683988	*S. alfalfae* ACCC40021
Geos33_Strep34	WP_098245470	*S. formicae* KY5
Geos34_Strep35	WP_087886527	*S. alboflavus* MDJK44
Geos35_Strep36	WP_040246537	*S. albus* BK3-25
Geos36_Strep37	WP_040246537_1	*S. albus* DSM 41398
Geos37_Strep38	WP_075986266	*S. sp.* FR-008
Geos38_Strep39	WP_003951048	*S. albus* J1074
Geos39_Strep40	WP_079055460	*S. albus* SM254
Geos40_Strep41	WP_030308315	*S. sp.* SM17
Geos41_Strep42	WP_095682396	*S. sp.* CLI2509
Geos42_Strep43	WP_044369842	*S. globisporus* TFH56
Geos43_Strep44	WP_058953825	*S. globisporus* C-1027
Geos44_Strep45	WP_075268060	*S. sp.* Tü 6075
Geos45_Strep46	WP_012382258	*S. griseus subsp. griseus* NBRC 13350
Geos46_Strep47	WP_083191937	*S. violaceoruber* S21
Geos47_Strep48	WP_053562493	*S. sp.* CFMR 7
Geos48_Strep49	WP_084996823	*S. sp.* S8
Geos49_Strep50	WP_015606689	*S. fulvissimus* DSM 40593
Geos50_Strep51	WP_014044184	*S. sp.* SirexAA-E
Geos51_Strep52	WP_103493052	*S. sp.* SM18
Geos52_Strep53	WP_014158016	*S. pratensis* ATCC 33331
Geos53_Strep54	WP_015575862	*S. sp.* PAMC26508
Geos54_Strep55	WP_078076350	*S. niveus* SCSIO 3406
Geos55_Strep56	ABY50951	*S. peucetius subsp. caesius* ATCC 27952
Geos56_Strep57	WP_005321403	*S. pristinaespiralis* HCCB 10218
Geos57_Strep58	WP_108147039	*S. lunaelactis* MM109
Geos58_Strep59	ANW21593	*S. clavuligerus* F613-1
Geos59_Strep60	WP_108908415	*S. sp.* HNM0039
Geos60_Strep61	WP_109297326	*S. spongiicola* HNM0071
Geos61_Strep62	WP_055645174	*S. venezuelae* ATCC 15439

Geos62_Strep63	WP_015031476	*S. venezuelae* NRRL B-65442
Geos63_Strep64	WP_041127665	*S. vietnamensis* GIM4.0001
Geos64_Strep65	WP_062757268	*S. sp.* WAC00288
Geos65_Strep66	BAU81127	*S. laurentii* ATCC 31255
Geos66_Strep67	WP_069975090	*S. rubrolavendulae* MJM4426
Geos67_Strep68	WP_078095378	*S. sp.* fd1-xmd
Geos68_Strep69	WP_075971290	*S. sp.* TN58
Geos69_Strep70	WP_081522109	*S. sp.* Sge12
Geos70_Strep71	WP_008743400	*S. sp.* Mg1
Geos71_Strep72	WP_030234522	*S. lavendulae subsp. lavendulae* CCM 3239
Geos72_Strep73	WP_084772522	*S. sp.* MOE7
Geos73_Strep74	WP_069572885	*S. lydicus* 103
Geos74_Strep75	WP_109891303	*S. sp.* NEAU-S7GS2
Geos75_Strep76	WP_083106597	*S. gilvosporeus* F607
Geos76_Strep77	WP_046926865	*S. lydicus* A02
Geos77_Strep78	WP_039641183	*S. sp.* 769
Geos78_Strep79	WP_079143472	*S. noursei* ATCC 11455
Geos79_Strep80	WP_038526177	*S. albulus* NK660
Geos80_Strep81	WP_037632171	*S. albulus* ZPM
Geos81_Strep82	WP_099016999	*S. malaysiensis* DSM 4137
Geos82_Strep83	WP_079258182	*S. autolyticus* CGMCC0516
Geos83_Strep84	WP_100806825	*S. sp.* M56
Geos84_Strep85	WP_014061818	*S. violaceusniger* Tü 4113
Geos85_Strep86	WP_078647520	*S. hygroscopicus* XM201
Geos86_Strep87	WP_014174668	*S. bingchenggensis* BCW-1
Geos87_Strep88	WP_087929697	*S. albireticuli* MDJK11
Geos88_Strep89	WP_030731770	*S. xiamenensis* 318
Geos89_Strep90	WP_086159477	*S. sp.* SCSIO 03032
Geos90_Strep91	WP_101425785	*S. sp.* CMB-StM0423
Geos91_Strep92	WP_052770207	*S. sp.* CNQ509
Geos92_Strep93	WP_014143690	*S. cattleya* NRRL 8057

Table S7: List of 2-methylisoborneol synthases used to build the DTL tree in Figure S7.

Abbreviation	Accession number	Species name
Strep1_Mib1	NP_733742	*S. coelicolor* A3(2)
Strep2_Mib2	WP_011031839	*S. lividans* TK24
Strep5_Mib3	WP_064725957	*S. parvulus* 2297
Strep6_Mib4	WP_063481016	*S. ambofaciens* DSM 40697
Strep7_Mib5	WP_053126184	*S. ambofaciens* ATCC 23877
Strep18_Mib6	WP_107448681	*S. sp.* P3
Strep24_Mib7	WP_069782778	*S. puniciscabiei* TW1S1
Strep27_Mib8	WP_020938197	*S. collinus* Tü 365
Strep28_Mib9	WP_067309437	*S. griseochromogenes* ATCC 14511(1)
Strep28_Mib10	WP_067310286	*S. griseochromogenes* ATCC 14511(2)
Strep29_Mib11	WP_064537133	*S. sp.* SAT1
Strep32_Mib12	WP_041668842	*S. scabiei* 87.22(1)
Strep32_Mib13	WP_037726550	*S. scabiei* 87.22(2)
Strep45_Mib14	WP_075263486	*S. sp.* Tü 6075
Strep46_Mib15	WP_012378420	*S. griseus subsp. griseus* NBRC 13350
Strep53_Mib16	WP_014157663	*S. pratensis* ATCC 33331
Strep54_Mib17	WP_015576150	*S. sp.* PAMC26508
Strep55_Mib18	WP_078079412	*S. niveus* SCSIO 3406
Strep59_Mib19	ANW17109	*S. clavuligerus* F613-1
Strep62_Mib20	WP_055645320	*S. venezuelae* ATCC 15439
Strep64_Mib21	WP_041132328_1	*S. vietnamensis* GIM4.0001
Strep65_Mib22	WP_062750724	*S. sp.* WAC00288
Strep66_Mib23	BAU87358	*S. laurentii* ATCC 31255
Strep68_Mib24	WP_078095811	*S. sp.* fd1-xmd
Strep69_Mib25	WP_075971735	*S. sp.* TN58
Strep70_Mib26	WP_081521483	*S. sp.* Sge12
Strep71_Mib27	WP_047960430	*S. sp.* Mg1
Strep72_Mib28	WP_078950304	*S. lavendulae subsp. lavendulae* CCM 3239
Strep73_Mib29	WP_084775022	*S. sp.* MOE7

Strep74_Mib30	WP_069571074	*S. lydicus* 103
Strep75_Mib31	WP_109889928	*S. sp.* NEAU-S7GS2
Strep76_Mib32	WP_083108965	*S. gilvosporeus* F607(2)
Strep76_Mib33	WP_083103453	*S. gilvosporeus* F607(1)
Strep77_Mib34	WP_046924697	*S. lydicus* A02(1)
Strep77_Mib35	WP_078984193	*S. lydicus* A02(2)
Strep78_Mib36	WP_039628838	*S. sp.* 769(1)
Strep78_Mib37	WP_078876140	*S. sp.* 769(2)
Strep79_Mib38	WP_079143205	*S. noursei* ATCC 11455
Strep80_Mib39	WP_038524797	*S. albulus* NK660
Strep81_Mib40	WP_020930496	*S. albulus* ZPM
Strep82_Mib41	WP_099012977	*S. malaysiensis* DSM 4137
Strep83_Mib42	WP_079256828	*S. autolyticus* CGMCC0516
Strep84_Mib43	WP_100807892	*S. sp.* M56
Strep85_Mib44	WP_014058647	*S. violaceusniger* Tü 4113
Strep86_Mib45	WP_078645903	*S. hygroscopicus* XM201
Strep87_Mib46	WP_043488086	*S. bingchenggensis* BCW-1
Strep91_Mib47	WP_101423704	*S. sp.* CMB-StM0423
Strep92_Mib48	WP_047016550	*S. sp.* CNQ-509

Table S8: List of *epi*-isozizaene synthases used to build the DTL tree in Figure S8.

Abbreviation	Accession number	Species name
Strep1_Epi1	NP_629369	*S. coelicolor* A3(2)
Strep2_Epi2	AIJ13444	*S. lividans* TK24
Strep3_Epi3	WP_061446904	*S. sp.* CCM MD2014
Strep4_Epi4	WP_078535684	*S. pactum* KLBMP 5084
Strep5_Epi5	WP_064731961	*S. parvulus* 2297
Strep6_Epi6	WP_079155896	*S. ambofaciens* DSM 40697
Strep7_Epi7	WP_079030788	*S. ambofaciens* ATCC 23877
Strep8_Epi8	WP_079160747	*S. pactum* ACT12
Strep9_Epi9	WP_047122496	*S. leeuwenhoekii* C34
Strep10_Epi10	WP_044388300	*S. cyaneogriseus subsp. noncyanogenus* NMWT 1
Strep11_Epi11	WP_029181723	*S. chartreusis* NRRL 3882
Strep12_Epi12	WP_107308381	*S. sp.* S10(2016)
Strep13_Epi13	OSC69340	*S. sp.* 4F(1)
Strep14_Epi14	WP_058917971	*S. sp.* 4F(2)
Strep14_Epi15	WP_099052949	*S. glaucescens* GLA.O
Strep15_Epi16	WP_110630412_1	*S. actuosus* ATCC 25421
Strep16_Epi17	WP_107416269	*S. sp.* CdTB01
Strep17_Epi18	WP_107406875	*S. lincolnensis* NRRL 2936
Strep18_Epi19	WP_107441985	*S. sp.* P3
Strep19_Epi20	WP_108710823	*S. sp.* 452
Strep30_Epi31	WP_107083301	*S. avermitilis* MA-4680
Strep31_Epi32	WP_099500021	*S. sp.* XZHG99
Strep33_Epi33	APY90801	*S. alfalfae* ACCC40021
Strep34_Epi34	WP_098244686	*S. formicae* KY5
Strep35_Epi35	WP_087885793_2	*S. alboflavus* MDJK44
Strep36_Epi36	WP_107071290	*S. albus* BK3-25
Strep37_Epi37	WP_107071290_2	*S. albus* DSM 41398
Strep38_Epi38	WP_030765460	*S. albus* SM254
Strep39_Epi39	WP_008415715	*S. albus* J1074

Strep40_Epi40	WP_030765460_2	*S. sp*. FR-008
Strep41_Epi41	AWL34654	*S. sp*. SM17
Strep42_Epi42	WP_095682130_1	*S. sp*. CLI2509

Table S9. Habitats of the *Streptomyces* species represented in the whole genome-based phylogenetic tree. The species are separated according to the three phylogenetic clades (green, blue and red) shown in the phylogenetic tree in Figure 1.

Organism name	Isolated from	Habitat	Ref	Terpene synthases*
S. coelicolor A3(2)	Soil	Terrestrial	[2]	● ○ ●
S. lividans TK24	Soil	Terrestrial	[3]	● ○ ●
S. sp. CCM_MD2014	Soil	Terrestrial	[4]	● ●
S. pactum KLBMP 5084	Halophyte plant endophyte	Terrestrial	[5]	● ●
S. parvulus 2297	Laboratory (derivative of *S. parvulus* ATCC 12434; soil)	Terrestrial	[6-7]	● ○ ● ●
S. ambofaciens DSM 40697	Soil	Terrestrial	[8]	● ○ ● ●
S. ambofaciens ATCC 23877	Soil	Terrestrial	[9]	● ○ ● ●
S. pactum ACT12	Soil	Terrestrial	[10-11]	● ● ●
S. leeuwenhoekii C34	Soil (desert)	Terrestrial	[12]	● ● ● ●
S. cyaneogriseus subsp. noncyanogenus NMWT 1	Soil (sand)	Terrestrial	[13]	● ● ● ●
S. chartreusis NRRL 3882	Soil	Terrestrial	[14]	● ● ● ●
S. sp. S10(2016) (*S. qaidamensis* S10(2016))	Soil (sand)	Terrestrial	[15]	● ● ●
S. sp. 4F	Saline lake	Aquatic	[16]	● ●● ●
S. glaucescens GLA.O	Soil	Terrestrial	[17]	● ● ● ●
S. actuosus ATCC 25421	Soil	Terrestrial	[18]	● ●
S. sp. CdTB01	Soil (heavy metals contaminated)	Terrestrial	[19]	● ●
S. lincolnensis NRRL 2936	Soil	Terrestrial	[20]	● ●
S. sp. P3	Potato scab diseased tubers	Terrestrial	[21]	● ○ ●
S. sp. 452 (*S. nigra* 452)	Mangrove soil	Aquatic	[22]	● ●
S. hygroscopicus subsp. jinggangensis 5008	Soil	Terrestrial	[23]	● ●
S. hygroscopicus subsp. jinggangensis TL01	Laboratory (derivative of strain 5008; soil)	Terrestrial	[24]	● ●
S. hygroscopicus subsp. limoneus KCTC 1717	Soil	Terrestrial	[25]	● ●
S. reticuli Tü 45	Soil	Terrestrial	[26]	● ●
S. puniciscabiei TW1S1	Soil (acidic)	Terrestrial	[27]	● ○ ● ●
S. pluripotens MUSC 137	Mangrove soil	Aquatic	[28]	● ●
S. pluripotens MUSC 135	Mangrove soil	Aquatic	[28]	● ●
S. collinus Tü 365	Soil	Terrestrial	[29]	● ○ ● ● ●

S. griseochromogenes ATCC 14511	Soil	Terrestrial	[30]	●	○● (grey)	● (red)		● (brown)
S. sp. SAT1	Plant endophyte	Terrestrial	[31]	●	○	● (red)		● (brown)
S. avermitilis MA-4680	Soil	Terrestrial	[32]	●		● (red)		● (orange)
S. sp. XZHG99 (*S. dengpaensis* XZHG99)	Desert soil	Terrestrial	[33]	●		● (red)	● (purple)	
S. scabiei 87.22	Plant pathogen	Terrestrial	[34]	●	○○			
S. alfalfae ACCC40021	Soil (alfalfa rhizosphere)	Terrestrial	[35]	●		● (red)		● (orange)
S. formicae KY5	Plant ant *Tetraponera penzigi*	Terrestrial	[36]	●		● (red)		
S. alboflavus MDJK44	Soil (peony rhizosphere)	Terrestrial	[37]	●		● (red)		
S. albus BK3-25	Soil	Terrestrial	[38]	●		● (red)		
S. albus DSM 41398	Soil	Terrestrial	[38]	●		● (red)		
S. sp. FR-008	Laboratory (derivative of *S. hygroscopicus* var. yingchengensis; soil)	Terrestrial	[39]	●		● (red)		
S. albus J1074 (*S. albidoflavus* J1074)	Laboratory (derivative of *S. albus* DSM 41398; soil)	Terrestrial	[40]	●		● (red)		
S. albus SM254	Soil (iron mine)	Terrestrial	[41]	●		● (red)		
S. sp. SM17	Sponges	Marine	[42]	●		● (red)		
S. sp. CLI2509	Bracket fungus	Terrestrial	[43]	●		● (red)		

Organism name	Isolated from	Habitat	Ref	Terpene synthases*				
S. globisporus THF56	Tomato flower	Terrestrial	[44]	●		● (light blue)	● (blue)	
S. globisporus C-1027	Soil	Terrestrial	[45]	●		● (light blue)	● (blue)	
S. sp. Tü 6075	Soil	Terrestrial	[46]	●	○	● (light blue)	● (blue)	
S. griseus subsp. griseus NBRC 13350	Soil	Terrestrial	[47]	●	○	● (light blue)	● (blue)	
S. violaceoruber S21	Seabed sludge	Marine	[48]	●		● (light blue)	● (blue)	
S. sp. CFMR 7	Rubber tree	Terrestrial	[49]	●		● (light blue)	● (blue)	
S. sp. S8	Soil (turfgrass)	Terrestrial	[50]	●		● (light blue)	● (blue)	
S. fulvissimus DSM 40593	unknown	unknown	[51]	●		● (light blue)	● (blue)	
S. sp. SirexAA-E	Woodwasp *Sirex noctilio*	Terrestrial	[52]	●		● (light blue)	● (blue)	
S. sp. SM18	Marine Sponge Haliclona simulans	Marine	[42]	●		● (light blue)	● (blue)	● (purple)
S. pratensis ATCC 33331	Soil	Terrestrial	[53]	●	○	● (light blue)		● (purple)
S. sp. PAMC 26508	Antarctic lichen *Cladonia borealis*	Terrestrial	[54]	●	○	● (light blue)		● (purple)
S. niveus SCSIO 3406	Deep sea sediment	Marine	[55]	●	● (grey)			
S. peucetius subsp. caesius ATCC 27952	Soil	Terrestrial	[56]	●				

S. pristinaespiralis HCCB 10218	Laboratory (derivative of *S. pristinaespiralis* ATCC 25486)	unknown	[57]	●
S. lunaelactis MM109	Cave moonmilk deposits	Terrestrial	[58]	●
S. clavuligerus F613-1	Laboratory (derivative of *S. clavuligerus* ATCC 27064; soil)	Terrestrial	[59-60]	● ○
S. sp. HNM0039	Marine sponge	Marine	[61]	●
S. spongiicola HNM0071	Marine sponge	Marine	[62]	●
S. venezuelae ATCC 15439	Soil	Terrestrial	[63]	● ○
S. venezuelae NRRL B-65442	Laboratory (origin unknown)	unknown	[64]	●
S. vietnamensis GIM4.0001	Soil (tropical forest)	Terrestrial	[65]	● ○
S. sp. WAC00288	Soil	Terrestrial	[66]	● ○
S. laurentii ATCC 31255	Soil	Terrestrial	[67]	● ○ ●
S. rubrolavendulae MJM4426	Soil	Terrestrial	[68]	● ●
S. sp. fd1-xmd	Soil	Terrestrial	[69]	● ○
S. sp. TN58	Soil	Terrestrial	[70]	● ○
S. sp. Sge12	Soil (forest)	Terrestrial	[71]	● ○
S. sp. Mg1	Soil	Terrestrial	[72]	● ○
S. lavendulae subsp. lavendulae CCM 3239	unknown	unknown	[73]	● ○
Organism name	Isolated from	Habitat	Ref	Terpene synthases*
S. sp. MOE7	Soil (agriculture)	Terrestrial	[74]	● ●
S. lydicus 103	Soil	Terrestrial	[75]	● ●
S. sp. NEAU-S7GS2	Soil	Terrestrial	[76]	● ●
S. gilvosporeus F607	Laboratory (derivative of *S. gilvosporeus* TCC 13326; soil)	Terrestrial	[77]	● ○●
S. lydicus A02	Soil	Terrestrial	[78]	● ○●
S. sp. 769	Soil	Terrestrial	[79]	● ○●
S. noursei ATCC 11455	Soil (dairy farm)	Terrestrial	[80]	● ●
S. albulus NK660	Soil	Terrestrial	[81]	● ●
S. albulus ZPM	Soil	Terrestrial	[82]	● ●
S. malaysiensis DSM 4137	Soil	Terrestrial	[83]	● ○ ● ●
S. autolyticus CGMCC 0516	Soil	Terrestrial	[84]	● ○ ● ●
S. sp. M56	Termites nest	Terrestrial	[85]	● ○ ● ●
S. violaceusniger Tü 4113	Soil	Terrestrial	[86]	● ○ ● ●

S. hygroscopicus XM201	Soil	Terrestrial	[87]	geosmin, 2-methylisoborneol-1, cyclooctat-9-en-7-ol, isoafricanol
S. bingchenggensis BCW-1	Soil	Terrestrial	[88]	geosmin, 2-methylisoborneol-1, isoafricanol, pentalenene
S. albireticuli MDJK11	Soil (peony rhizosphere)	Terrestrial	[37]	geosmin
S. xiamenensis 318	Mangrove sediment	Aquatic	[89]	geosmin
S. sp. SCSIO 03032	Marine sediment	Marine	[90]	geosmin
S. sp. CMB-StM0423	Beach sand	Terrestrial	[91]	geosmin, 2-methylisoborneol-3
S. sp. CNQ-509	Marine sediment	Marine	[92]	geosmin, 2-methylisoborneol-3
S. cattleya NRRL 8057 = DSM 46488	Soil	Terrestrial	[93]	geosmin

* Terpene synthases: ● geosmin, ○ 2-methylisoborneol-1, ● 2-methylisoborneol-2 ● 2-methylisoborneol-3 ● epi-isozizaene ● 7-epi-α-eudesmol ● epi-cubenol ● caryolan-1-ol ● cyclooctat-9-en-7-ol ● isoafricanol ● pentalenene ● α-amorphene

The pattern of selection in terpene synthase family

To understand the selective pressures that have shaped the distribution of the terpene synthases, we tested evolutionary models implemented in HyPhy, individually with the geosmin, 2-MIB and *epi*-isozizaene synthases. After discarding redundant sequences, gene sequences encoding geosmin, 2-MIB and *epi*-isozizaene synthases were aligned. Recombinant regions and regions with long stretches of gaps were detected using RDP (recombinant detection program, [94]) and further trimmed from the sequence alignments. The selection intensity was analysed separately for the three different gene categories. For *epi*-isozizaene, 27% of sites (116 out of 419 sites) in the coding sequence alignment had dN/dS values lower than 1.0 indicating that they are under negative or purifying selection (p-value threshold of 0.01). This suggests that substitutions in 27% of sites in the coding sequence of the *epi*-isozizaene synthase gene are being purged and not being maintained. Similarly, 17% of sites (96 out of 559 sites) in the 2-MIB group were also found under negative or purifying selection. On the contrary, for the geosmin synthase gene, a small percentage of the sites were found to be under positive selection. Some sites (54 out of 962, 5.6%) are under positive or diversifying selection (substitutions in these sites are being maintained) and only 0.5% of sites are under negative selection (5 out of 962) (Figure S2). Other evolutionary models (implemented in HyPhy like FUBAR and ABSREL) were also tested. For gene categories *epi*-isozizaene and 2-MIB, FUBAR detected 156 and 155 sites respectively under negative or purifying selection (with posterior probability of 0.99). In the case of geosmin synthase, FUBAR detected 92 sites under positive or diversifying selection (with posterior probability of 0.99). This suggests that the domains of the geosmin synthase and the function they determine are targeted by selection. A remarkable cluster of codon positions in the N-terminal

domain of geosmin synthases were detected under positive selection. In accordance with this, the N-terminal part of geosmin synthase was shown to be highly conserved among *Streptomyces* and essential for the conversion of FPP to germacradienol and germacrene [95].

Phylogeny of terpene synthases does not correspond to species-level taxonomy

NOTUNG analyses [96] were performed to reconcile an associate tree with a reference tree. Under the cost matrix for duplications, transfers, and losses as used by TreeFix, NOTUNG recovered most parsimonious scenarios with 25 putative transfers and 13 corresponding losses in the geosmin synthase category. However, NOTUNG failed to infer any events in *epi*-isozizaene and 2-MIB categories. This can be explained by inherent topological errors in the maximum-likelihood (ML) trees for *epi*-isozizaene and 2-MIB synthases. Treefix-DTL (duplication-transfer-loss) was used to correct topological inconsistencies in all available terpene synthase trees, including the geosmin synthase tree. The DTL-reconciliation problem is typically solved in a parsimony framework, where costs are assigned to DTL events and the goal is to find reconciliation with minimum total cost [97]. For the individual categories, treefix-DTL minimised the DTL cost and generated trees with minimum reconciliation cost among all the associated trees that have likelihood statistically equivalent to that of the ML trees (Figures S6-S8). Accordingly, the subsequent NOTUNG analysis successfully recovered a minimal number of events in all the three categories. NOTUNG inferred a total of 19 transfers and 10 losses while reconciling the geosmin synthase tree with the *Streptomyces* species whole genome-based tree. Similarly, the number of transfer/loss in *epi*-isozizaene-species tree reconciliation

were 7/4 and with 2-MIB the counts were 22/11. The reconciled trees are available in the Supporting Information (Figures S6–S8).

References

1. Emms, D. M.; Kelly, S. *Genome Biol.* **2015,** *16*, 157.2. Bentley, S. D.; Chater, K. F.; Cerdeno-Tarraga, A. M.; Challis, G. L.; Thomson, N. R.; James, K. D.; Harris, D. E.; Quail, M. A.; Kieser, H.; Harper, D.; Bateman, A.; Brown, S.; Chandra, G.; Chen, C. W.; Collins, M.; Cronin, A.; Fraser, A.; Goble, A.; Hidalgo, J.; Hornsby, T.; Howarth, S.; Huang, C. H.; Kieser, T.; Larke, L.; Murphy, L.; Oliver, K.; O'Neil, S.; Rabbinowitsch, E.; Rajandream, M. A.; Rutherford, K.; Rutter, S.; Seeger, K.; Saunders, D.; Sharp, S.; Squares, R.; Squares, S.; Taylor, K.; Warren, T.; Wietzorrek, A.; Woodward, J.; Barrell, B. G.; Parkhill, J.; Hopwood, D. A. *Nature* **2002,** *417* (6885), 141-147.

3. Ruckert, C.; Albersmeier, A.; Busche, T.; Jaenicke, S.; Winkler, A.; Friethjonsson, O. H.; Hreggviethsson, G. O.; Lambert, C.; Badcock, D.; Bernaerts, K.; Anne, J.; Economou, A.; Kalinowski, J. *J. Biotechnol.* **2015,** *199*, 21-2.

4. Mariita, R. M.; Bhatnagar, S.; Hanselmann, K.; Hossain, M. J.; Korlach, J.; Boitano, M.; Roberts, R. J.; Liles, M. R.; Moss, A. G.; Leadbetter, J. R.; Newman, D. K.; Dawson, S. C. *Genome Announc* **2015,** *3* (6).

5. Qin, S.; Feng, W. W.; Wang, T. T.; Ding, P.; Xing, K.; Jiang, J. H. *Plant and Soil* **2017,** *416* (1-2), 117-132.

6. Nishizawa, T.; Miura, T.; Harada, C.; Guo, Y.; Narisawa, K.; Ohta, H.; Takahashi, H.; Shirai, M. *Genome Announc* **2016,** *4* (4).

7. Kirby, R.; Hopwood, D. A. *Microbiology* **1977,** *98* (1), 239-252.

8. Thibessard, A.; Leblond, P. *Genome Announcements* **2016,** *4* (3), e00470-16.

9. Thibessard, A.; Haas, D.; Gerbaud, C.; Aigle, B.; Lautru, S.; Pernodet, J. L.; Leblond, P. *J. Biotechnol.* **2015,** *214*, 117-8.

10. Zhao, J. X., Quan-Hong; Wang, Ling-Na; Duan, Chun-Mei; Xue, Lei; Mao, Ning. *Chinese Journal of Eco-Agriculture* **2011,** *19*, 394-398.

11. Cao, S.; Wang, W.; Wang, F.; Zhang, J.; Wang, Z.; Yang, S.; Xue, Q. *Environ Sci Pollut Res Int* **2016,** *23* (15), 14898-907.

12. Busarakam, K.; Bull, A. T.; Girard, G.; Labeda, D. P.; van Wezel, G. P.; Goodfellow, M. *Antonie Van Leeuwenhoek* **2014,** *105* (5), 849-61.

13. Wang, H.; Li, C.; Zhang, B.; He, H.; Jin, P.; Wang, J.; Zhang, J.; Wang, X.; Xiang, W. *J. Biotechnol.* **2015,** *204*, 1-2.

14. Doroghazi, J. R.; Ju, K. S.; Brown, D. W.; Labeda, D. P.; Deng, Z.; Metcalf, W. W.; Chen, W.; Price, N. P. *J. Bacteriol.* **2011,** *193* (24), 7021-2.

15. Zhang, B.; Tang, S.; Chen, X.; Zhang, G.; Zhang, W.; Chen, T.; Liu, G.; Li, S.; Dos Santos, L. T.; Castro, H. C.; Facey, P.; Hitchings, M.; Dyson, P. *J. Antibiot. (Tokyo)* **2018,** *71* (10), 880-886.

16. Cornell, C. R.; Marasini, D.; Fakhr, M. K. *Front. Microbiol.* **2018,** *9*, 2282.

17. Ortseifen, V.; Winkler, A.; Albersmeier, A.; Wendler, S.; Puhler, A.; Kalinowski, J.; Ruckert, C. *J. Biotechnol.* **2015,** *194*, 81-3.

18. Dosch, D. C. S., William R; Floss, Heinz G. *Biochem. Biophys. Res. Commun.* **1988,** *156* (1), 517-523.

19. Zhou, G.; Yang, H.; Zhou, H.; Wang, C.; Fu, F.; Yu, Y.; Lu, X.; Tian, Y. *J. Biotechnol.* **2016,** *229*, 42-3.

20. Meng, S. C., Enhancement of antibiotic productions by engineered nitrateutilization in actinobacteria. GenBank, 2016.

21. Kang, M. K. P., Duck Hwan *Korean J. Microbiol.* **2018,** *54* (2), 158-160.

22. Chen, C., *Streptomyces nigra* sp. nov. is a Novel Actinobacterium Isolated from Mangrove Soil and a Potential Inhibitor of Human Cancer Cell Lines. GenBank, 2018.

23. Yu, Y.; Bai, L.; Minagawa, K.; Jian, X.; Li, L.; Li, J.; Chen, S.; Cao, E.; Mahmud, T.; Floss, H. G.; Zhou, X.; Deng, Z. *Appl. Environ. Microbiol.* **2005,** *71* (9), 5066-76.

24. Zhou, X.; Wu, H.; Li, Z.; Zhou, X.; Bai, L.; Deng, Z. *Metab. Eng.* **2011,** *13* (6), 768-76.
25. Lee, S. H.; Choe, H.; Bae, K. S.; Park, D. S.; Nasir, A.; Kim, K. M. *J. Biotechnol.* **2016,** *219*, 1-2.
26. Wibberg, D.; Al-Dilaimi, A.; Busche, T.; Wedderhoff, I.; Schrempf, H.; Kalinowski, J.; Ortiz de Orue Lucana, D. *J. Biotechnol.* **2016,** *222*, 13-4.
27. Kim, M.-K. K., S.B., *Streptomyces puniciscabiei* strain:TW1S1 Genome sequencing and assembly. GenBank, 2016.
28. Lee, L. H.; Zainal, N.; Azman, A. S.; Eng, S. K.; Ab Mutalib, N. S.; Yin, W. F.; Chan, K. G. *Int. J. Syst. Evol. Microbiol.* **2014,** *64* (Pt 9), 3297-306.
29. Wolf, H. Z., H. *Arch. Mikrobiol.* **1972,** *83*, 147-154.
30. Wu, L.; Chen, G.; Feng, G. *J. Biotechnol.* **2017,** *249*, 16-19.
31. Dou, G., Complete genome of endophytic *Streptomyces* sp. GenBank, 2016.
32. Ikeda, H.; Ishikawa, J.; Hanamoto, A.; Shinose, M.; Kikuchi, H.; Shiba, T.; Sakaki, Y.; Hattori, M.; Omura, S. *Nat. Biotechnol.* **2003,** *21* (5), 526-31.
33. Li, Y.; Li, Y.; Wang, L. W.; Bao, J. *Int. J. Syst. Evol. Microbiol.* **2018,** *68* (10), 3322-3326.
34. Bignell, D. R. D. S., Ryan F.; Huguet-Tapia, J. C.; Chambers, A. H.; Parry, R. J.; Loria, R. *Mol. Plant-Microbe Interact.* **2010,** *23* (2), 161-175.
35. Gu, J., GenBank, 2016.
36. Holmes, N. A.; Devine, R.; Qin, Z.; Seipke, R. F.; Wilkinson, B.; Hutchings, M. I. *J. Biotechnol.* **2018,** *265*, 116-118.
37. Wang, C.; Wang, Y.; Ma, J.; Hou, Q.; Liu, K.; Ding, Y.; Du, B. *Biomed. Res. Int.* **2018,** *2018*, 2419686.
38. Lu, C.; Zhang, X.; Jiang, M.; Bai, L. *Metab. Eng.* **2016,** *35*, 129-137.
39. Liu, Q.; Xiao, L.; Zhou, Y.; Deng, K.; Tan, G.; Han, Y.; Liu, X.; Deng, Z.; Liu, T. *Synth. Syst. Biotechnol.* **2016,** *1* (3), 207-214.
40. Chater, K. F. W., L. C. *J. Bacteriol.* **1976,** *128* (2), 644-650.
41. Badalamenti, J. P.; Erickson, J. D.; Salomon, C. E. *Genome Announc.* **2016,** *4* (2).
42. Jackson, S. A.; Crossman, L.; Almeida, E. L.; Margassery, L. M.; Kennedy, J.; Dobson, A. D. W. *Mar. Drugs* **2018,** *16* (2).
43. Wyche, T. P.; Ruzzini, A. C.; Schwab, L.; Currie, C. R.; Clardy, J. *J. Am. Chem. Soc.* **2017,** *139* (37), 12899-12902.
44. Cho, G., Complete genome sequence of *Streptomyces globisporus* TFH56 isolated from tomato flower. GenBank, 2018.
45. Wang, L.; Wang, S.; He, Q.; Yu, T.; Li, Q.; Hong, B. *J. Bacteriol.* **2012,** *194* (15), 4144.
46. Schimana, J. G., K.; Holtzelb, A.; Schmidb, D. G.; Sussmuth, R.; Muller, J., Pukall, R.; Fiedler, H. P. *J. Antibiot.* **2002,** *55* (6), 565-570.
47. Ohnishi, Y.; Ishikawa, J.; Hara, H.; Suzuki, H.; Ikenoya, M.; Ikeda, H.; Yamashita, A.; Hattori, M.; Horinouchi, S. *J. Bacteriol.* **2008,** *190* (11), 4050-60.
48. Fu, J.; Zhong, C.; Zhao, Z.; Zong, G.; Cao, G. *Genome Data* **2017,** *12*, 116-117.
49. Nanthini, J.; Chia, K. H.; Thottathil, G. P.; Taylor, T. D.; Kondo, S.; Najimudin, N.; Baybayan, P.; Singh, S.; Sudesh, K. *J. Biotechnol.* **2015,** *214*, 47-8.
50. Cho, G.; Kim, J.; Park, C. G.; Nislow, C.; Weller, D. M.; Kwak, Y. S. *Open Biol.* **2017,** *7* (7).
51. Myronovskyi, M.; Tokovenko, B.; Manderscheid, N.; Petzke, L.; Luzhetskyy, A. *J. Biotechnol.* **2013,** *168* (1), 117-8.
52. Adams, A. S.; Jordan, M. S.; Adams, S. M.; Suen, G.; Goodwin, L. A.; Davenport, K. W.; Currie, C. R.; Raffa, K. F. *ISME J.* **2011,** *5* (8), 1323-31.

53. Rong, X.; Doroghazi, J. R.; Cheng, K.; Zhang, L.; Buckley, D. H.; Huang, Y. *Syst. Appl. Microbiol.* **2013,** *36* (6), 401-7.
54. Shin, S. C. P., H.; Genome sequence of *Streptomyces* sp. PAMC26508, isolated from Antarctic lichen *Cladonia borealis*. https://www.pacb.com/wp-content/uploads/Poster_GenomeSequencing_EndosymbioticBacteriaStreptomyces_AntarcticLichen_SMRTTechnology.pdf.
55. Song, Y.; Huang, H.; Chen, Y.; Ding, J.; Zhang, Y.; Sun, A.; Zhang, W.; Ju, J. *J. Nat. Prod.* **2013,** *76* (12), 2263-8.
56. Stutzman-Engwall, K. J. H., R. *Proc. Natl. Acad. Sci. USA* **1989,** *86*, 3135-3139.
57. Huang, H.; Zheng, G.; Jiang, W.; Hu, H.; Lu, Y. *Acta Biochim. Biophys. Sin. (Shanghai)* **2015,** *47* (4), 231-43.
58. Maciejewska, M.; Pessi, I. S.; Arguelles-Arias, A.; Noirfalise, P.; Luis, G.; Ongena, M.; Barton, H.; Carnol, M.; Rigali, S. *Antonie Van Leeuwenhoek* **2015,** *107* (2), 519-31.
59. Cao, G.; Zhong, C.; Zong, G.; Fu, J.; Liu, Z.; Zhang, G.; Qin, R. *Genome Announc.* **2016,** *4* (5).
60. Li, J.; Zhao, Z.; Zhong, W.; Zhong, C.; Zong, G.; Fu, J.; Cao, G. *3 Biotech.* **2018,** *8* (11), 472.
61. Huang, X. Z., S., Complete genome sequence of sponge-derived *Streptomyces* sp. HNM0039. 2018.
62. Huang, X.; Zhou, S.; Huang, D.; Chen, J.; Zhu, W. *Int. J. Syst. Evol. Microbiol.* **2016,** *66* (2), 738-743.
63. He, J.; Sundararajan, A.; Devitt, N. P.; Schilkey, F. D.; Ramaraj, T.; Melancon, C. E., 3rd. *Genome Announc.* **2016,** *4* (3).
64. Som, N. F.; Heine, D.; Holmes, N. A.; Munnoch, J. T.; Chandra, G.; Seipke, R. F.; Hoskisson, P. A.; Wilkinson, B.; Hutchings, M. I. *Front. Microbiol.* **2017,** *8*, 1145.
65. Deng, M. R.; Guo, J.; Ma, L. Y.; Li, Y. X.; Feng, G. D.; Mo, C. Y.; Zhu, H. H. *J. Biotechnol.* **2015,** *200*, 6-7.
66. Ho, L. K. N., J. R., *Streptomyces* sp. WAC00288 complete genome. 2018.
67. Doi, K.; Fujino, Y.; Nagayoshi, Y.; Ohshima, T.; Ogata, S. *Genome Announc.* **2016,** *4* (3).
68. Cheng, J.; Park, S. B.; Kim, S. H.; Yang, S. H.; Suh, J. W.; Lee, C. H.; Kim, J. G. *J. Appl. Microbiol.* **2016,** *120* (4), 975-85.
69. Yu, Y.; Tang, B.; Dai, R.; Zhang, B.; Chen, L.; Yang, H.; Zhao, G.; Ding, X. *Appl. Microbiol. Biotechnol.* **2018,** *102* (6), 2621-2633.
70. Najah, S.; Chong, T. M.; Gerbaud, C.; Chan, K.-G.; Mellouli, L.; Pernodet, J.-L. *Genome Announc.* **2017,** *5* (34), e00828-17.
71. Xu, J.; Xu, M.; Liu, K.; Peng, Q.; Tao, M. *Genome Announc.* **2017,** *5* (21), e00415-17.
72. Hoefler, B. C.; Konganti, K.; Straight, P. D. *Genome Announc.* **2013,** *1* (4).
73. Busche, T.; Novakova, R.; Al'Dilaimi, A.; Homerova, D.; Feckova, L.; Rezuchova, B.; Mingyar, E.; Csolleiova, D.; Bekeova, C.; Winkler, A.; Sevcikova, B.; Kalinowski, J.; Kormanec, J.; Ruckert, C. *Genome Announc.* **2018,** *6* (9).
74. Elnahas, M. O.; De Leon, K. B.; Amin, M. A.; Hussein, M. M. D.; Ali, A. E.; Wall, J. D. *Genome Announc.* **2017,** *5* (22).
75. Jia, N.; Ding, M. Z.; Luo, H.; Gao, F.; Yuan, Y. J. *Sci. Rep.* **2017,** *7*, 44786.
76. Huang, S.-X. W., Xiang-Jing; Yan, Yijun; Wang,Ji-Dong; Zhang, Ji; Liu, Chong-Xi; Xiang, Wen-Sheng; Shen, Ben. *Org.Lett.* **2012,** *14* (5), 1254-1257.
77. Zong, G.; Zhong, C.; Fu, J.; Zhao, Z.; Cao, G. *Genome Announc.* **2018,** *6* (1), e01402-17.
78. Wu, H.; Liu, W.; Shi, L.; Si, K.; Liu, T.; Dong, D.; Zhang, T.; Zhao, J.; Liu, D.; Tian, Z.; Yue, Y.; Zhang, H.; Xuelian, B.; Liang, Y. *Sci. Rep.* **2017,** *7* (1), 9114.

79. Du, Q. W., Q.; Li, Q.; Wang, L.; Zhang, Z.; Ren, J.; Wang, J.; Wang, M.; , Complete Genome Sequence of *Streptomyces gongzhulingensis*. 2012.
80. Ruckert, C.; Albersmeier, A.; Winkler, A.; Zotchev, S.; Kalinowski, J.; , Complete genome sequence of *Streptomyces noursei* ATCC 11455, a producer of the medically important antifungal antibiotic nystatin. 2015.
81. Gu, Y.; Yang, C.; Wang, X.; Geng, W.; Sun, Y.; Feng, J.; Wang, Y.; Quan, Y.; Che, Y.; Zhang, C.; Gong, T.; Zhang, W.; Gao, W.; Zuo, Z.; Song, C.; Wang, S. *Genome Announc.* **2014,** *2* (3).
82. Wang, L.; Gao, C.; Tang, N.; Hu, S.; Wu, Q. *Sci. Rep.* **2015,** *5*, 9201.
83. Rabe, P.; Samborskyy, M.; Leadlay, P. F.; Dickschat, J. S. *Org. Biomol. Chem.* **2017,** *15* (11), 2353-2358.
84. Yin, M.; Jiang, M.; Ren, Z.; Dong, Y.; Lu, T. *J. Biotechnol.* **2017,** *252*, 27-31.
85. Kim, K. H.; Ramadhar, T. R.; Beemelmanns, C.; Cao, S.; Poulsen, M.; Currie, C. R.; Clardy, J. *Chem. Sci.* **2014,** *5* (11), 4333-4338.
86. Höltzel, A.; Kempter, C.; Metzger, J. W.; Jung, G. *J. Antibiot.* **1998,** *51* (8), 699-707.
87. Wang, X.; Ning, X.; Zhao, Q.; Kang, Q.; Bai, L. *Biotechnol. J.* **2017,** *12* (11).
88. Wang, X. J.; Yan, Y. J.; Zhang, B.; An, J.; Wang, J. J.; Tian, J.; Jiang, L.; Chen, Y. H.; Huang, S. X.; Yin, M.; Zhang, J.; Gao, A. L.; Liu, C. X.; Zhu, Z. X.; Xiang, W. S. *J. Bacteriol.* **2010,** *192* (17), 4526-7.
89. Xu, M. J.; Wang, J. H.; Bu, X. L.; Yu, H. L.; Li, P.; Ou, H. Y.; He, Y.; Xu, F. D.; Hu, X. Y.; Zhu, X. M.; Ao, P.; Xu, J. *Sci. Rep.* **2016,** *6*, 18977.
90. Ma, L.; Zhang, W.; Zhu, Y.; Zhang, G.; Zhang, H.; Zhang, Q.; Zhang, L.; Yuan, C.; Zhang, C. *Appl. Microbiol. Biotechnol.* **2017,** *101* (15), 6123-6136.
91. Khalil, Z. G.; Cruz-Morales, P.; Licona-Cassani, C.; Marcellin, E.; Capon, R. J. *ISME J.* **2018**.
92. Ruckert, C.; Leipoldt, F.; Zeyhle, P.; Fenical, W.; Jensen, P. R.; Kalinowski, J.; Heide, L.; Kaysser, L. *J. Biotechnol.* **2015,** *216*, 140-1.
93. Li, P.; Tai, C.; Deng, Z.; Gan, J.; Oggioni, M. R.; Ou, H. Y. *Sci Rep* **2016,** *6*, 32047.
94. Martin, D. P.; Murrell, B.; Golden, M.; Khoosal, A.; Muhire, B. *Virus Evol.,* **2015,** *1*, vev003.
95. Jiang, J.; He, X.; Cane, D. E. *Nat. Chem. Biol.,* **2007,** *3*, 711-715.
96. Darby, C. A.; Stolzer, M.; Ropp, P. J.; Barker, D.; Durand, D. *Bioinformatics,* **2017,** *33*, 640-649.
97. Bansal, M. S.; Wu, Y.-C.; Alm, E. J.; Kellis, M. *Bioinformatics*, **2015,** *31*, 1211-1218.
